# Alterations in mitochondria and cellular senescence in aged sEH null female kidneys

**DOI:** 10.1007/s11357-025-01814-3

**Published:** 2025-07-26

**Authors:** Ala Yousef, Liye Fang, Mobina Heidari, Andy Huang, Patrick Kondraciuk, Kristen A. Yee, Michael Mengel, John M. Seubert

**Affiliations:** 1 Faculty of Pharmacy and Pharmaceutical Sciences, University of Alberta, Edmonton, AB Canada; 2 Department of Pharmacology, Faculty of Medicine and Dentistry, University of Alberta, Edmonton, AB Canada; 3 Department of Laboratory Medicine and Pathology, University of Alberta, Edmonton, Canada

**Keywords:** Soluble epoxide hydrolase, Mitochondria, Senescence, Aging, Kidney, Female

## Abstract

Age-related structural and functional deterioration of the kidneys is common among elderly individuals and contributes to increased mortality and morbidity. Mitochondrial dysfunction and cellular senescence are two hallmarks of aging that drive a progressional renal decline; however, the underlying molecular mechanisms and endogenous regulators behind these processes remain incompletely understood. The metabolism of polyunsaturated fatty acids by CYP450 enzymes produces numerous bioactive lipid mediators that can be further metabolized by soluble epoxide hydrolase (sEH) and microsomal epoxide hydrolase (mEH) into diol metabolites, often with reduced biological effects. The objective of this study was to assess renal mitochondrial alterations and cellular senescence in young and aged wild-type (WT) and sEH-deficient (sEH null) female mice. We found aged sEH null mice exhibited better physiological health, as reflected by lower frailty index scores and reduced circulating levels of GDF-15 levels, creatinine, and urea nitrogen. Notably, the expression of both sEH and mEH was significantly elevated in aged WT kidneys, accompanied by increased expression of the kidney injury marker (*Kim-1*) and evidence of structural abnormalities. In contrast, sEH deletion attenuated the age-related upregulation of senescence markers (p53, p21, p16) and SASP components (MCP-1, IL-1β, and caspase-1), as well as the inflammatory zBP1 expression and downstream interferons. Additionally, sEH deletion preserved age-related disruption of mitochondrial dynamics, content, and respiratory function. Together, these data suggest that sEH deletion confers renoprotective effects in aging, characterized by improved systemic health, reduced renal injury and inflammation as preserves mitochondrial integrity and function.

**Supplementary Information:**

The online version contains supplementary material available at 10.1007/s11357-025-01814-3.

## Introduction

Aging increases susceptibility to a variety of diseases, including cardiovascular, neurodegenerative, and renal disorders [1, 2]. As the body ages, decline in renal function involves molecular, structural, and physiological changes that can exacerbate systemic conditions such as hypertension, diabetes, or kidney disorders [3, 4]. Age-related physiological changes can compromise the kidney’s ability to repair itself, making older adults more susceptible to acute kidney injury, chronic diseases, and numerous renal complications [5]. Various cellular processes and molecular pathways take part in the complex process of aging [6]. Senescence of renal tubular cells has a pivotal role in advancing kidney aging and the onset of age-related renal fibrosis. Senescent cells not only lose their regenerative capabilities but also adopt a senescence-associated secretory phenotype (SASP), which promotes inflammation and fibrogenesis [7]. Eliminating these senescent cells has been shown to alleviate kidney aging phenotypes and maintain renal function [8]. However, there is a need to obtain a deeper understanding of the mechanisms underlying renal cellular senescence to provide insight into the aging kidney. Various theories have been proposed to explain aging mechanisms, including oxidative stress, disrupted protein homeostasis, DNA damage, and mitochondrial dysfunction [9, 10]. The critical role mitochondria have in regulating cellular homeostasis and energy production contributes to their importance in the aging process [11]. Mitochondrial dysfunction-associated senescence (MiDAS) can induce premature aging, characterized by excessive ROS production, impaired mitochondrial dynamics, reduced mitophagy, accumulation of defective proteins, and impaired electron transport chain function—contributing to an accelerated aging process [12]. Consistently, research demonstrates mitochondrial dysfunction is a critical driver in the progression of kidney aging [5, 13].

The beneficial effects of dietary N-3 and N-6 polyunsaturated fatty acids (PUFAs) are, in part, mediated by their metabolism into epoxylipid derviatives via cytochrome P450 (CYP) enzymes. These bioactive lipid mediators contribute to homeostatic regulation and exert protective effects; for instance, epoxyeicosatrienoic acids (EETs) have been shown to reduce inflammation and kidney fibrosis in animal models of chronic kidney diseases [14–16]. However, the epoxylipids are readily metabolized by epoxide hydrolase enzymes, particularly soluble epoxide hydrolase (sEH or *Ephx2*) and microsomal epoxide hydrolase (mEH or *Ephx1*) to diol products [17]. Several studies correlate increased sEH expression to adverse effects associated with the aging process in numerous organs like the heart [18, 19], brain [20], intestines [21], and liver [22]. Experimental evidence demonstrates deleting or inhibiting sEH may provide a protective response in aged-related cardiovascular, neurodegenerative, and inflammatory diseases [18, 23–25]. Most evidence supporting the protective effects of sEH inhibition comes from studies in young rodent models. For example, podocyte-specific sEH disruption protects against acute renal injury through the preservation of EET levels and the attenuation of NF-κB signaling activation [26]. Similarly, global sEH knockout mitigates glomerular injury and renal inflammation in deoxycorticosterone (DOCA)-salt-induced hypertension models [27]. In diabetic mouse models, sEH inhibition lessens tubular injury [28] and reduces overall kidney damage [29]. While these findings highlight a role of sEH in renal pathology, its contribution to kidney aging—particularly in relation to mitochondrial dysfunction and cellular senescence—remains poorly understood. In the present study, we examine the role of sEH in the context of renal aging using a genetic model of sEH (*Ephx2*) deletion and their wild-type littermates, assessing both young and aged mice. Our findings demonstrate that sEH deletion confers protective effects against age-associated renal decline by attenuating cellular senescence and senescence-associated secretory (SASP) markers, while preserving mitochondrial health.

## Methods

### Animals

Experiments were performed using young (4-month-old) and aged (24-month-old) female mice, which reflect human aging stages of 20–30 and 56–69 years, respectively [30]. Mice with global deletion of sEH (sEH null) were compared to wild-type (WT) littermate controls [31]. All animal experiments were approved by the University of Alberta Health Sciences Welfare Committee and followed Canadian Council on Animal Care’s Guide to the Care and Use of Experimental Animals [32]. Mice were housed at the University of Alberta’s Health Sciences Laboratory Animal Services facility in well-ventilated cages. Mice were kept in conditions of constant humidity, temperature, and light/dark cycles of 12-h:12-h with unrestricted access to a standard chow diet and water.

### Frailty index assessment

Frailty index tools were used to assess the health status as described [19, 33, 34]. Briefly, 31 physiological parameters were rated on a 3-point scale, yielding a total impairment score. Mean and standard deviation calculations were performed for body weight and body temperature for each group. Higher frailty index scores reflect increased physiological impairment and reduced overall health status. Experimenters performing frailty index assessments were blinded to mouse genotype and age.

### Tissue collection

Mice were given a lethal dose of sodium pentobarbital (100 mg/kg ip). Mouse kidneys were harvested and flash frozen in liquid nitrogen. Frozen kidneys were ground, homogenized, and subcellular fractionated as previously described [35]. Tissue homogenate was processed with subcellular fractionation into plasma membrane, mitochondrial, and cytosolic fractions for immunoblotting analysis. Briefly, frozen kidney tissues were ground in a ceramic mortar on dry ice and then homogenized in ice-cold homogenization buffer (20 mmol/L Tris–HCL, 1 mmol/L EDTA, and 250 mmol/L sucrose added on the day of the experiment, pH 7.0) with Pierce Protease and Phosphatase Inhibitor (PIA32959, Thermo Fisher Scientific). Samples were first centrifuged at 800 × g for 10 min at 4 °C to separate the cellular debris, and the collected supernatant was then centrifuged at 10,000 × g for 20 min. The pellet was resuspended in homogenization buffer to obtain a mitochondrial-enriched fraction. The supernatant was ultracentrifuged at 105,000 × g for 60 min and the subsequent supernatant was used as the cytosolic fraction. Plasma membrane–associated proteins in the debris pellet were solubilized with homogenization buffer containing 8 M urea [36].

### Real-time quantitative polymerase chain reaction

RNA was extracted from frozen kidney tissue using TRIzol RNA isolation reagent according to the manufacturer’s instructions (1,556,026, Thermo-Fisher Scientific). Concentration and purity analysis of RNA samples was performed using a UV–Vis spectrophotometer (NanoDrop; Thermo-Fisher Scientific). Complementary DNA (cDNA) was synthesized from RNA (1 µg) for each sample using a High-Capacity cDNA Reverse Transcriptase Kit (4,368,814; Thermo-Fisher Scientific). DNA (1 µg) was used for DNA analyses. Briefly, real-time PCR was performed using SYBR Green Reagent (A46109; Thermo-Fisher Scientific) to assess the expression changes in *Mcp1*,* IL-1β*,* p16*,* p21*,* p53*,* Kim-1*,* cGAS*,* zBP1*,* Ifit-1*,* Ifnb1*, and *Gapdh*; primers used are listed in Table S1. *Gapdh* was used as a housekeeping gene to compare relative mRNA levels between samples. Relative gene expression was calculated using 2^^−ΔΔCt^ method based on cycle threshold (Ct) values [37].

### Mitochondrial and cytosolic mtDNA

Mitochondrial DNA (mtDNA) copy number was used as a marker of mitochondrial content [38]. Total DNA was extracted from frozen kidney tissues, and qPCR was performed to assess the relative abundance of mitochondrial NADH-ubiquinone oxidoreductase chain 1 (Nd1) to nuclear DNA hexokinase-2 (Hk2) (expressed as mtDNA/nDNA). In another separate experiment aimed at evaluating cytosolic mtDNA levels, a marker of mitochondrial damage, a constant concentration 1 µg/µL of cytosolic fractions of kidney tissues was used and the relative expression of *Nd1* normalized to *18S rRNA* was calculated. No mitochondrial contamination was detected in the cytosolic fractions, and no cytosolic contamination was detected in the mitochondrial fractions (Figure S1). DNA extraction was performed using DNeasy tissue kit (69,504, Qiagen) according to the manufacturer’s protocol.

### Immunoblotting

Protein concentrations of subcellular fractions were determined using a Bradford protein assay (500–0002, Bio-Rad). Aliquots for gel electrophoresis were prepared, containing 35 µg protein, Laemmli buffer (1,610,747, Bio-Rad), and 2-mercaptoethanol (MER002; BioShop Canada). Protein was resolved by electrophoresis on (4–15%) SDS–polyacrylamide gels and transferred onto polyvinylidene difluoride (PVDF) membranes (1,620,177, BioRad). Immunoblots were probed with antibodies against sEH (1:1,000,10,833–1-AP, Proteintech), mEH (1:2000, sc135984, Santa Cruz), α-tubulin (1:1000, ab4074, Abcam), IL-1β (1:500, ab9722, Abcam), Caspase-1 (1:1000, ab179515, Abcam), MFN-2 (1:1000, cs9482, Cell Signaling) DRP-1 (1:1000, sc-271583, Santa Cruz), phosphorylated caveolin-1 (Y-14) (1:1000, cs3251S, Cell Signaling), caveolin-1 (1:1000, cs3267S, Cell Signaling), Parkin (1:1000, cs21325, Cell Signaling), PINK-1 (1:1000, ab23707, Abcam), VDAC1 (1:2000, ab14734, Abcam), and GAPDH (1:2,000, cs5174S, Cell Signaling). All primary incubations were followed with a horse radish peroxidase-conjugated goat anti-rabbit or anti-mouse IgG polyclonal antibody (1:2000, cs7074S and cs7076S, Cell Signaling). SuperSignal West PicoPLUS Chemilumesescent Substrate (Cat. No. A38556) and the Bio-Rad ChemiDoc Imaging System were used for visualization. Quantification of signal intensities was performed with ImageJ software (National Institutes of Health) to obtain protein expression levels, normalized to an appropriate control for differences content.

### Plasma creatinine, urea nitrogen, and GDF-15 quantification

Blood obtained from the portal vein was collected in an EDTA-coated tube and centrifugation at 2000 × g for 10 min at 4 °C. Plasma was isolated and promptly frozen in liquid nitrogen and stored at − 80 °C. Upon analysis, the collected plasma samples were thawed on ice, and creatinine and urea nitrogen levels were assessed using colorimetric assay kits (ab65340, Abcam and EIABUN, Thermo Fisher Scientific) according to the manufacturer’s instructions. Growth differentiation factor 15 (GDF-15) levels were measured using a mouse GDF-15 ELISA kit (RDR-GDF15-Mu, Reddot Biotech), following the manufacturer’s instructions.

### Enzyme activity assays

Kidney tissues were homogenized using ice-cold homogenization buffer, without protease or phosphatase inhibitors, and underwent subsequent subcellular fractionation. Caspase-1 was assessed in cytosolic fractions using the fluorogenic substrates Ac-YVAD-AMC (ALX-260–024-M005, Enzo Life Sciences). Briefly, 30 μl of cytosolic fractions was incubated at 37 °C for 30 min with the substrates in a 30 μl of working solution of assay buffer (20 mM HEPES, pH 7.4, 40 mM NaCl, 0.2% CHAPS, 0.4 mM EDTA, 4 mM DDT, and 4% glycerol). Caspase activity was proportional to the generation of fluorescent signal due to cleavage and release of free fluorescent 7-amino-4-methyl coumarin (AMC) from the caspase substrate. AMC concentrations for each sample were determined using a linear standard curve with free AMC and samples were normalized to their respective protein concentrations to obtain caspase activity as picomoles of AMC produced per microgram of protein per minute. Fluorescent signal was detected using a fluorescence plate reader using 380-nm excitation and 460-nm emission wavelengths. Citrate synthase (CS) and complex I enzyme activities were assessed spectrophotometrically in mitochondrial enriched fractions as previously described [39]. Enzymatic activity was normalized to protein concentration and expressed as nmol/min/mg protein.

### Histology and immunofluorescence staining

Fresh kidney tissues were fixed in 10% buffered formalin and processed in paraffin blocks. Kidney sections were cut into 5-μm-thick sections and stained with hematoxylin and eosin (H&E), as well as periodic acid-schiff (PAS) (*n* = 1 per group). Histological staining was performed by HistoCore facility at the Alberta Diabetes Institute, University of Alberta. For the immunofluorescence staining, fresh kidney tissues were sagittally sectioned and cryopreserved in optimal cutting temperature (OCT) solution and cut into 5-μm-thick sections. Sections were incubated with blocking buffer containing (5% goat serum) at room temperature then incubated with primary anti-p21 (1:100, ab109199, Abcam) or anti-CD68 + (1:1000, ab125212, Abcam) polyclonal antibodies overnight at 4 °C in a humidified chamber. Next, slides were washed and then incubated with the corresponding secondary antibody (Alexa Flour™ 568 goat anti-rabbit immunoglobulin G (H + L); A11011, Thermo Fisher Scientific) for 60 min at room temperature in the dark. Next, the slides were mounted with ProLongTM Gold anti-fade Mountant with 4′,6-diamidino-2-phenylindole (DAPI; P36935, Thermo Fisher Scientific). Sections were visualized using Zeiss Axio Observer Z1 widefield epifluorescence microscope and images were captured and analyzed using ZEN 2 Imaging Software. Quantification of immunofluorescence images was performed by an independent blinded observer. To assess protein expression, random fields were selected, and individual cells positive for p21 or CD68 were counted based on DAPI staining. The number of positive cells was normalized to the total number of DAPI-positive cells and expressed as a percentage per field.

### High-resolution mitochondrial respirometry

After mouse euthanisation, kidneys were harvested, collected in ice-cold phosphate buffered saline (PBS), and, then, freshly homogenized using a Teflon-glass pestle homogenizer with mitochondrial respiration medium MiR05 (pH 7.1, 0.5 mM EGTA, 3 mM MgCl2, 60 mM potassium lactobionic acid, 20 mM taurine, 10 mM KH2PO4, 20 mM HEPES, 110 mM d-sucrose, 1 g/L fatty acid-free bovine serum albumin (BSA), dissolved in molecular-grade H_2_O). A constant homogenate concentration (0.5 mg/mL) was added to the 2 mL respirometric chambers of Oroboros-O2k high-resolution respirometer (OROBOROS Instruments, Innsbruck, Austria). After sample addition to the chambers, (i) addition of complex I substrates 10 mM glutamate and 2 mM malate; (ii) addition of 2.5 mM adenosine diphosphate (ADP) to stimulate complex I-mediated oxidative phosphorylation (OXPHOS); (iii) addition of oligomycin 0.5 mM to inhibit the ATP‐dependent respiration; (iv) 0.1 μM step-wise titrations with FCCP until reaching a maximal noncoupled respiration rate; (v) addition of 0.5 μM rotenone to inhibit complex I mediated respiration to achieve residual oxygen consumption (ROX state). Oxygen consumption rate (OCR) was measured and obtained by subtracting ROX respiration from all respiration values and normalizing to homogenate concentration [40, 41].

### Statistics

Statistical analyses were performed using GraphPad Prism 9 software. Results are presented as mean ± SEM and statistical tests and parameters used are stated in figure legends. Briefly, two-way ANOVA followed by Tukey’s multiple comparison test and unpaired *t*-test was performed for mitochondrial respiration data. Statistical significance was determined at *p* < 0.05.

## Results

### Age-related decline in kidney function correlates with increased epoxide hydrolase expression

**Fig. 1:**
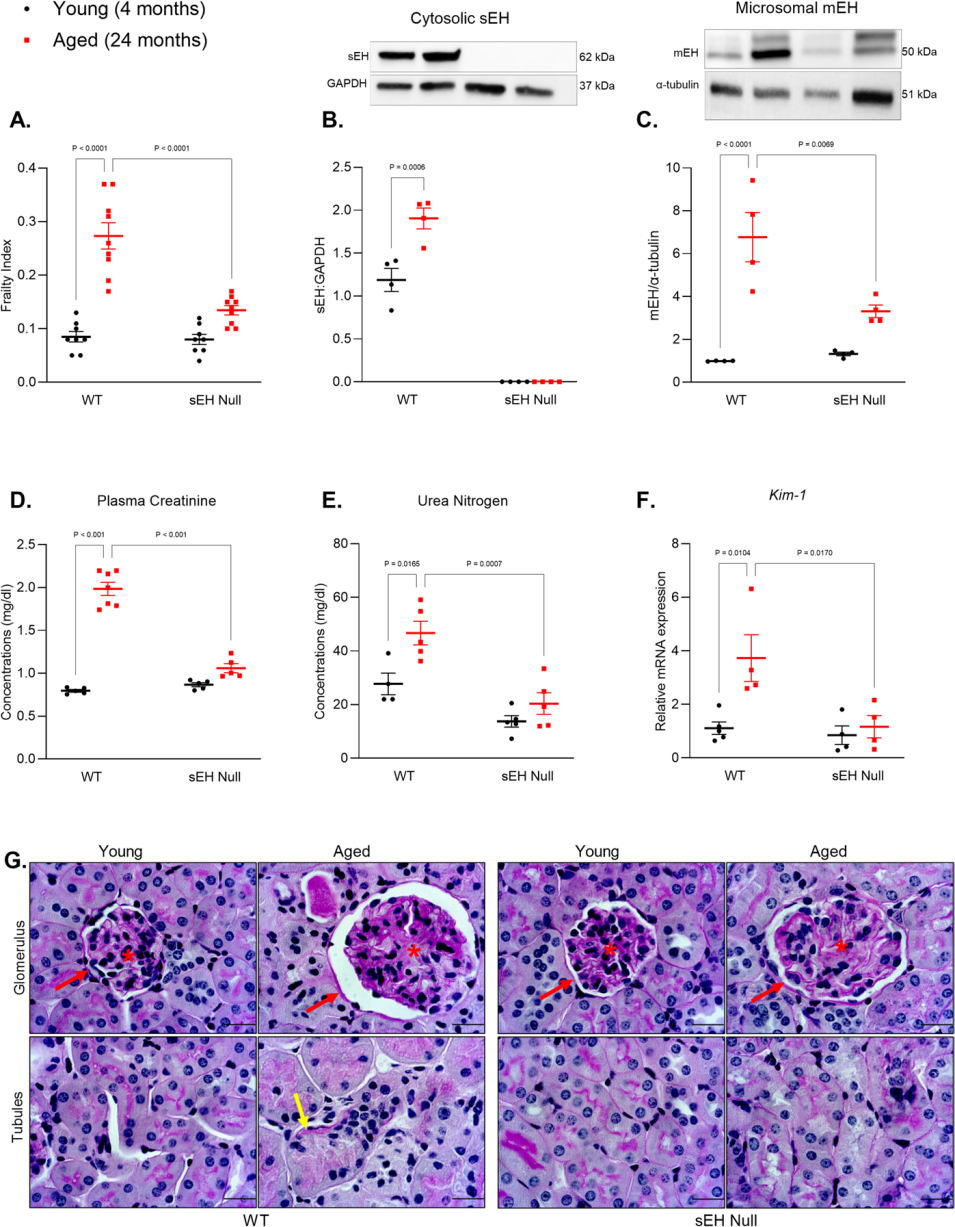
**A** Level of physiological impairment assessed by frailty index score in young and aged WT and sEH null mice (*n* = 8–9). **B** Protein expression of soluble epoxide hydrolase (sEH) relative to GAPDH expression in the cytosolic fraction (*n* = 4). **C** Protein expression of microsomal epoxide hydrolase (mEH) relative to α-tubulin in the microsomal fraction (*n* = 4). **D** Plasma creatinine concentration (*n* = 5–7). **E** Plasma urea nitrogen levels (*n* = 4–5). **F** Gene expression of kidney injury marker (*Kim-1*) relative to *Gapdh* as a house keeping gene (*n* = 4–5). **G** Representative images of periodic acid-Schiff (PAS)stained sections of young and aged WT and sEH null kidneys showing glomeruli (star) with mesangial hypercellularity in aged animals, Bowman’s capsule (red arrow), and acute tubular injury (yellow arrow). Scale bars = 20 μm. Data represented as mean ± SEM, *p* < 0.05. Statistical analysis was done using two-way ANOVA with Tukey’s post hoc test

To quantify and assess the accumulation of age-related health deficits, a frailty index was taken at both 4 and 24 months of age in each individual animal. There was a marked increase in the frailty index observed in WT compared to sEH null mice, reflecting a more significant deterioration in health status in aged WT mice (Fig. 1A). Increased sEH protein expression has been found in multiple aged organs such as the brain [20], heart [18, 19], intestines [21], and liver [22], in both human and murine models. In the current study, we observed a significant increased expression of sEH in the kidneys of aged (24 months) female WT mice (Fig. 1B). Similarly, microsomal epoxide hydrolase (mEH) expression was significantly increased in aged mice but was greater in WT compared to sEH null kidneys (Fig. 1C). Increased levels of circulating plasma creatinine, urea nitrogen, and gene expression of *Kim-1*, a common marker of kidney injury, showed a significant increase in aged WT mice suggesting a reduced kidney function (Fig. 1D–F). Representative PAS-stained kidney sections indicated structural alterations in aged WT mice, including enlarged glomeruli with thickened basement membranes and expanded mesangial areas showing segmentally increased cellularity. Few globally sclerosed glomeruli, focal interstitial inflammation, and focal features of acute tubular injury are seen in aged WT mice compared to young counterparts (Fig. 1G, S2). These features are suggestive of age-associated renal changes. Together, these results show that sEH protein expression is significantly increased in the aged WT mice, associated with increased injury markers and reduced kidney function.

### Genetic deletion of sEH attenuates senescence markers in aged kidneys

**Fig. 2:**
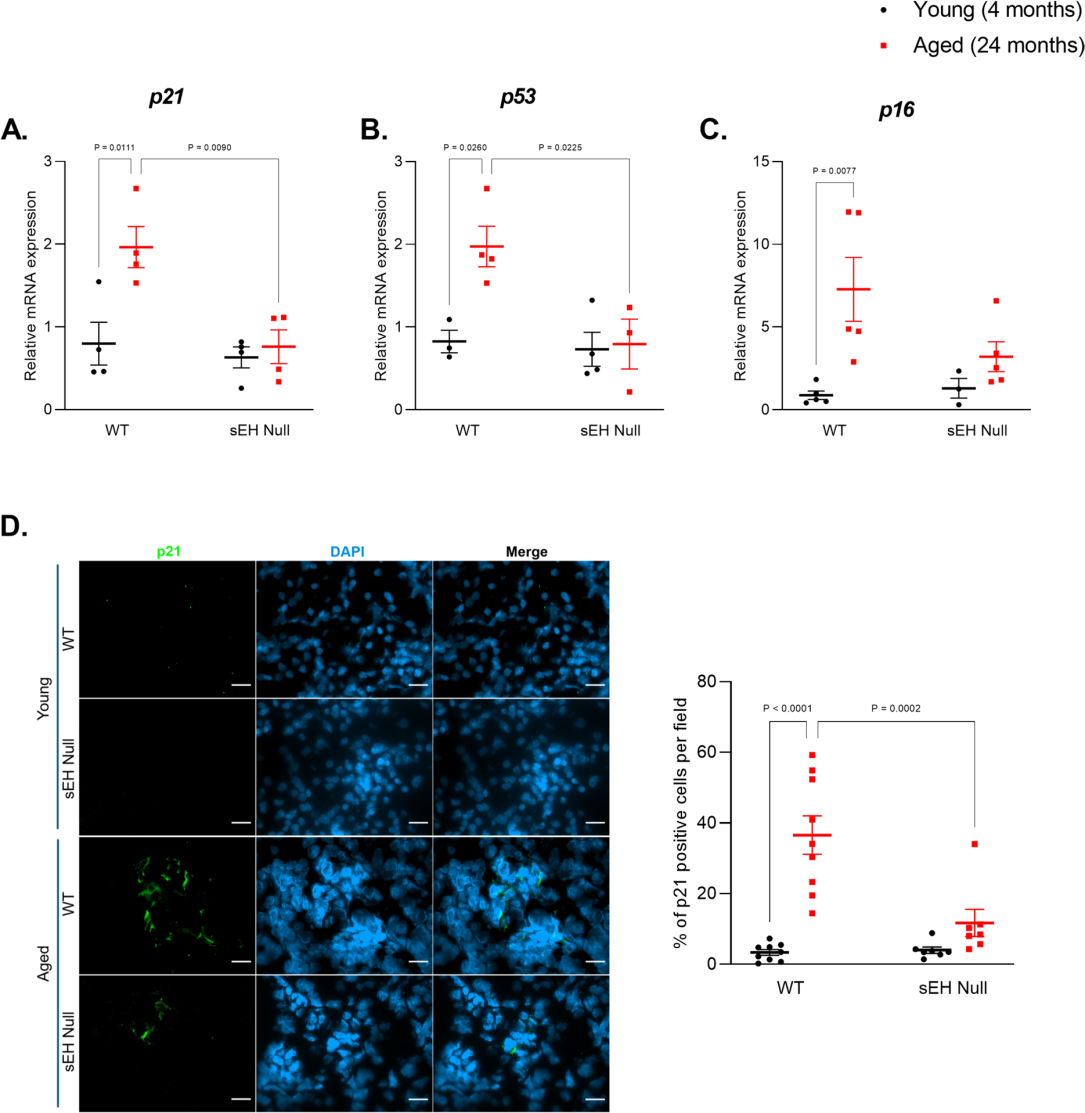
Measurement of senescent markers in young and aged kidney tissues from WT and sEH null mice. **A**–**C** Relative gene expression of *p21*, *p53*, and *p16* relative to the housekeeping gene *Gapdh* gene (*n* = 3–5). **D** Representative images of immunofluorescence staining of p21 in kidney tissue, and quantification of p21-positive cells. Scale bars = 50 μm. Data represented as mean ± SEM, *p* < 0.05. Statistical analysis was done using two-way ANOVA with Tukey’s post hoc test

Senescence is a key biological driver of aging, particularly at the cellular and tissue levels; however, our understanding of the role sEH has remained limited. We further studied the potential role of sEH in the aging process by comparing the expression of main senescent markers in young (4 months) and aged mice (24 months). Aged WT mice exhibited increased expression of multiple senescent markers, including *p21*, *p53*, and *p16* (Fig. 2A–C) which is consistent with previous human and animal studies showing the accumulation of the senescence regulators [6, 42]. Interestingly, genetic deletion of sEH blocked the upregulation of senescent markers in aged kidneys (Fig. 2A–C). Further evaluation of p21 protein levels in the kidneys was conducted using immunofluorescence staining. The renal protein expression of p21 was significantly higher in aged WT compared to sEH null counterparts (Fig. 2D).

### Genetic deletion of sEH attenuates SASP components in aged kidneys

**Fig. 3:**
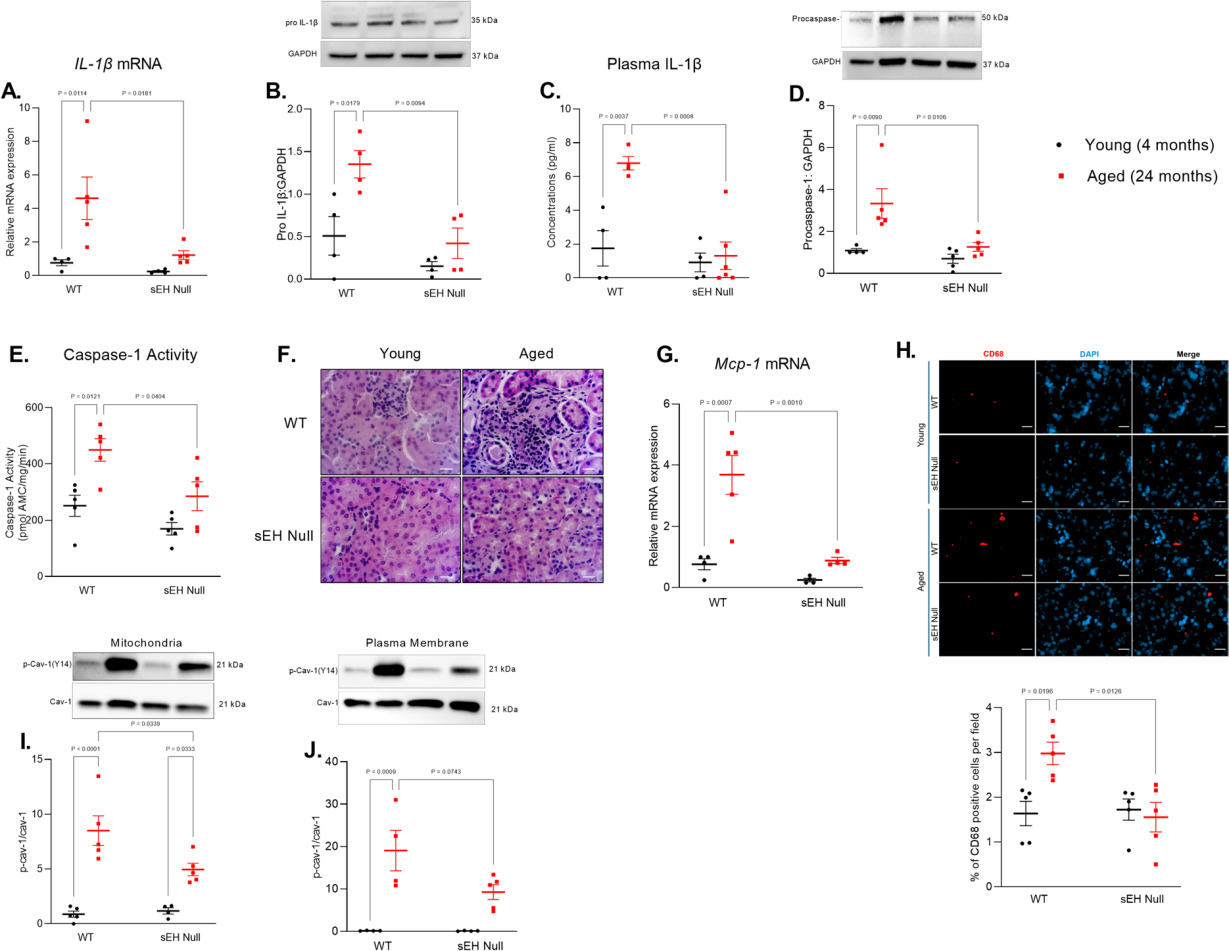
SASP and inflammatory markers in young and aged kidney tissues from WT and sEH null mice. **A** Relative gene expression of *IL-1β* to the housekeeping gene *Gapdh* (*n* = 4–5). **B** Protein expression of pro-IL-1β in the cytosol fraction of kidney tissues. **C** Plasma IL-1β protein concentration (*n* = 4–6). **D** Protein expression of pro-caspase-1 in the cytosol fraction of obtained from kidney tissues. **E** Cytosolic caspase-1 activity in kidney tissues (*n* = 5). **F** Representative images of hematoxylin and eosin (HE)-stained kidney sections of young and aged WT and sEH null kidneys. Scale bars = 200 μm. **G** Relative gene expression of *Mcp-1* to the housekeeping gene *Gapdh* (*n* = 4). **H** Representative images and quantification of CD68-positive cells. Scale bars = 50 μm. **I** Protein expression of phosphorylated Cav-1 (TYR-14) to total Cav-1 in mitochondrial enriched fractions. **J** Protein expression of phosphorylated Cav-1 (TYR-14) to total Cav-1 in plasma membrane (*n* = 4–5). Data represented as mean ± SEM, *p* < 0.05. Statistical analysis was done using two-way ANOVA with Tukey’s post hoc test

The observation that senescence markers are mainly increased in aged WT kidney suggests a detrimental role for sEH. Senescent cells are characterized by the secretion of several molecules, grouped under the name of SASP [43]. Several components of SASP known to play a potential role in aged tissues, such as IL-1β and MCP-1 [44]. A significant increase in *IL-1β* gene expression and pro IL-1β protein levels was observed in aged WT mice (Fig. 3A, B). As well, plasma levels of IL-1β were significantly increase aged WT mice but not in sEH null mice (Fig. 3C). Consistent with elevated IL-1β, we observed a marked increase in renal procaspase-1 expression and caspase-1 activity (Fig. 3D, E). Representative H&E-stained kidney sections suggested an interstitial inflammatory infiltrate in aged WT mice compared to young counterparts. This apparent inflammatory response appeared reduced in kidneys from sEH null mice (Fig. 3F). MCP-1 is a chemokine involved in recruitment immune cells, notably monocytes, and considered part of the SASP. Significant differences were observed in *MCP-1* gene expression in aged WT while no differences were observed in sEH null kidneys (Fig. 3G). Next, we investigated the recruitment of macrophages by assessing CD68^+^ protein expression. Results show increased CD68^+^ in aged WT kidneys compared to their young kidneys (Fig. 3H). Studies have linked caveolin-1 (cav-1) to aging and senescence [45–47], where phosphorylated cav-1 plays a role in promoting SASP driving chronic inflammation in aging tissues. Evidence suggests phosphorylated Cav-1 may potentially exacerbate mitochondrial dysfunction to drive aging and cellular senescence. Immunoblotting data demonstrates a dramatic age-related increase in phosphorylated Cav-1 in the plasma membrane and mitochondrial enriched fractions in aged kidneys (Fig. 3I, J), which was significantly higher in WT mice.

### Genetic deletion of sEH attenuates age-related disruption to mitochondria

**Fig. 4:**
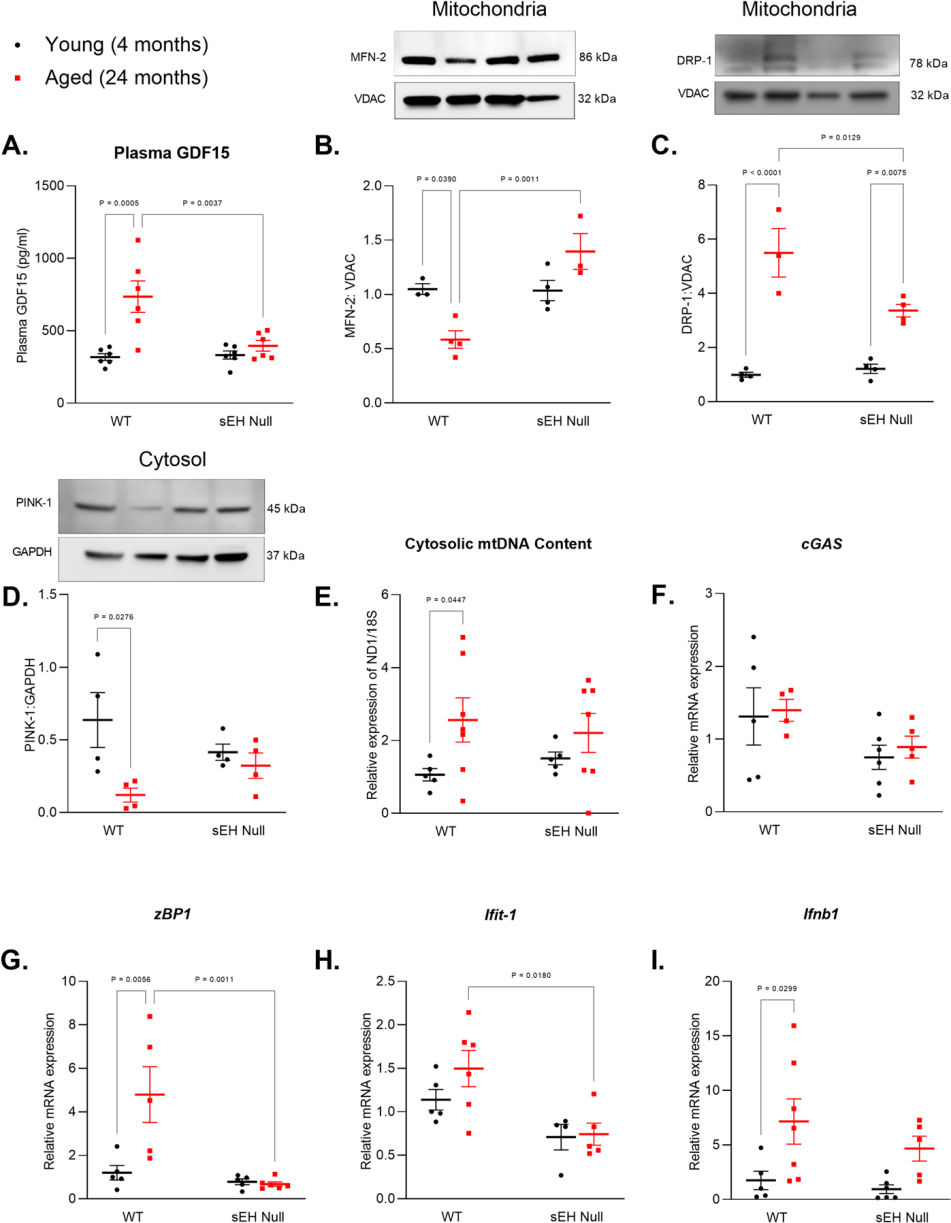
Markers of mitochondrial damage and disrupted dynamics in young and aged kidney from WT and sEH null mice. **A** Plasma GDF-15 protein levels. **B** Protein expression of MFN-2 in mitochondrial enriched fractions. **C** Protein expression of DRP-1 relative to VDAC-1 in mitochondrial enriched fraction. **D** Protein expression of PINK-1 in the cytosol fraction (*n* = 4). **E** Cytosolic mtDNA content expressed as relative gene expression of mitochondrial NADH dehydrogenase 1 Nd1 to 18S rRNA. **F**–**I** Relative gene expression of *cGAS*,* zBP1*, *Ifit-1*, and *Ifnb1* relative to the housekeeping gene *Gapdh* (*n* = 4–7). Data represented as mean ± SEM, *p* < 0.05 (*n* = 3–7). Statistical analysis was done using two-way ANOVA with Tukey’s post hoc test

GDF-15 is a member of the TGF-β cytokine superfamily and is expressed in multiple organs, including the kidneys, heart, skeletal muscles, and lungs [48, 49]. Evidence has linked GDF-15 with aging, mitochondrial dysfunction, and in the progression of age-related organ decline [50]. Plasma analyses showed elevated GDF-15 protein levels in aged WT mice compared to their young counterparts, whereas aged sEH null mice had significantly lower levels (Fig. 4A). Given that sEH deletion appeared to reduce mitochondrial stress, we next examined changes in markers of mitochondrial dynamics. A significant reduction in MFN-2, a protein involved in mitochondrial fusion, was observed in aged WT kidneys, whereas MFN-2 levels remained unchanged in aged sEH null mice (Fig. 4B). In contrast, levels of DRP-1, a mediator of mitochondrial fission, were markedly increased in aged kidneys, with significantly higher expression in WT mice compared to sEH null (Fig. 4C). Into further assess mitochondrial quality control, we examined mitophagy markers and found that PINK-1 protein levels were decreased exclusively in aged WT kidneys, while sEH deletion preserved PINK1 expression with age (Fig. 4D). Together, these findings indicate that sEH deletion supports the maintenance of mitochondrial homeostasis during aging by modulating mitochondrial dynamics and preserving mitophagic capacity. In contrast, altered expression of key proteins involved in mitochondrial balance, potentially contributing to the accumulation of dysfunctional mitochondria [51]. As an indicator of mitochondrial damage, we next assessed cytosolic mtDNA levels. A significant increase in cytosolic mtDNA was observed in aged WT kidneys, whereas no such elevation was detected in aged sEH null mice (Fig. 4E). To assess downstream signaling associated with cytosolic DNA sensing, we measured mRNA expression of components of the innate immune response, including *cGAS*,* zBP1*,* lfit-1*, and *Ifnb1*. While *cGAS* expression remained unchanged across groups (Fig. 4F), aged sEH null kidneys exhibited reduced expression of *zBP1* and inflammatory interferon-related genes (*lfit-1* and *Ifnb1*) compared to aged WT controls (Fig. 4G–I). Thus, suggesting sEH deletion mitigates age-related mitochondrial damage and dampens downstream inflammatory signaling mediated by cytosolic DNA sensors.

**Fig. 5:**
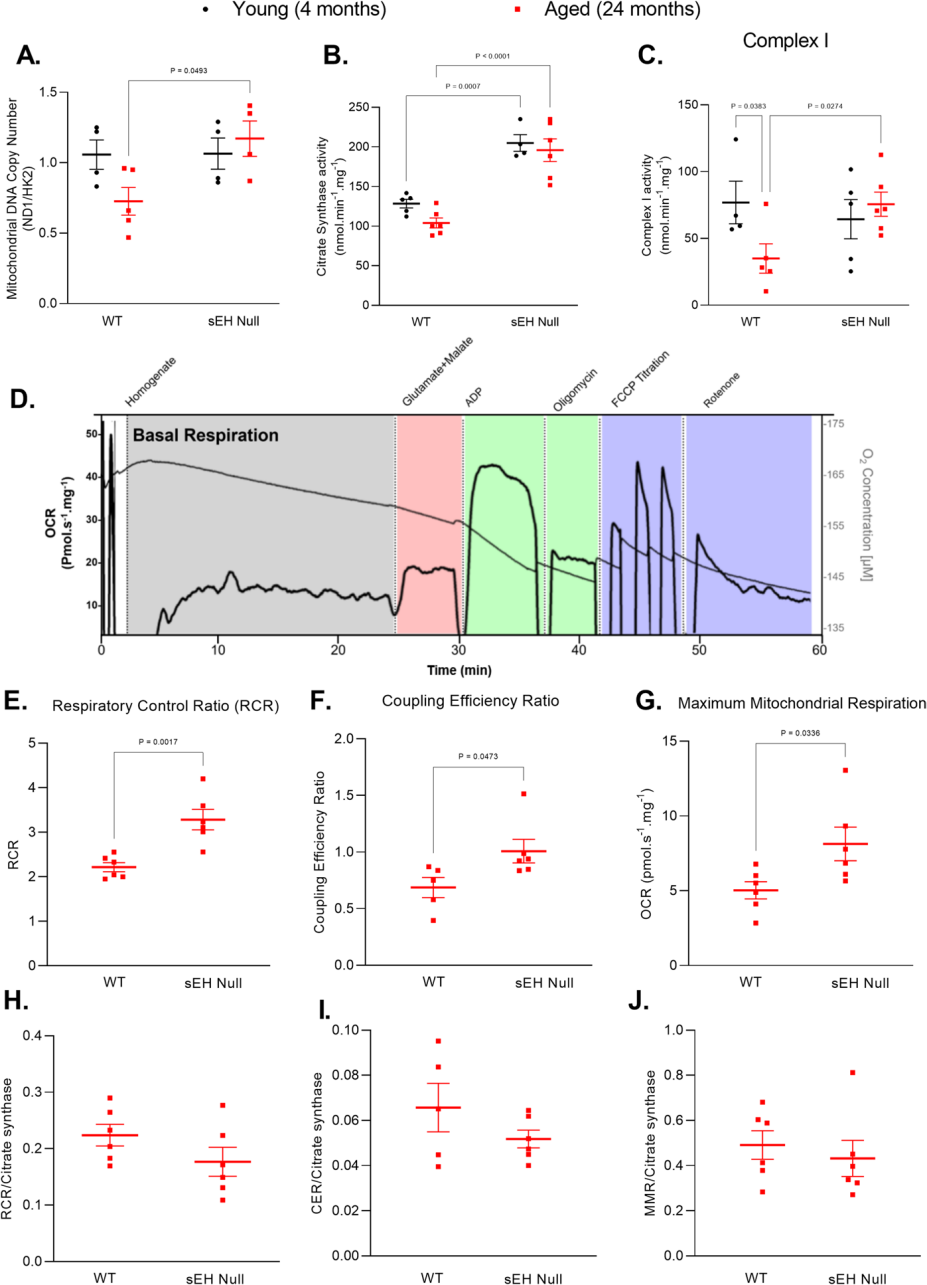
Mitochondrial content and function in young and aged kidneys from WT and sEH null mice. **A** Mitochondrial DNA copy number calculated by the ratio of mouse mitochondrial NADH dehydrogenase 1 (*Nd1*) to nuclear hexokinase (*Hk2*). **B** Citrate synthase activity in mitochondrial enriched fractions of kidney tissues. **C** Complex I activity in mitochondrial enriched fractions of kidney tissues. Data represented as mean ± SEM, *p* < 0.05. Statistical analysis was done using two-way ANOVA with Tukey’s post hoc test. Mitochondrial complex I respiration in aged WT and sEH null kidneys. **D** Representative time course of oxygen consumption rate (OCR) of kidney homogenate showing the administration of glutamate (10 mM) and malate (2 mM), ADP (2.5 mM), oligomycin (0.5 mM), carbonyl cyanide p‐trifluoro‐methoxyphenyl hydrazone (FCCP) (0.1 μM), and rotenone (0.5 μM). **E** Respiratory control ratio (RCR). **F** Coupling efficiency ratio (CER). **G** Maximum mitochondrial respiration (MMR). **H** Ratio of RCR/citrate synthase activity. **I** Ratio of CER/citrate synthase activity. **J** MMR/citrate synthase activity. Data represented as mean ± SEM, *p* < 0.05 (*n* = 4–6). Statistical analysis was done using unpaired *t*-test

Building on our findings that sEH deletion alleviates mitochondrial damage and suppresses inflammatory signaling in aged kidneys, we next investigated whether these protective effects were associated with improved mitochondrial health. The protective effect of sEH deletion has been linked to delayed progression of various diseases, in part through the preservation of mitochondrial function [24, 52–55]. As mitochondrial dysfunction can trigger the initiation of senescence [12, 56], we tested whether the anti-senescence effect associated with the sEH deletion correlated with better mitochondrial integrity in the kidney. We assessed measured mitochondrial DNA (mtDNA) copy number and citrate synthase activity as indicators of mitochondrial content. Aged WT mice exhibited a significant decline in mtDNA copy number compared to aged sEH null mice (Fig. 5A), accompanied by reductions in citrate synthase (Fig. 5B) and complex I activities (Fig. 5C). These deficits were attenuated in sEH null mice, suggesting preserved mitochondrial content.

To further explore the aging effect on the mitochondrial respiratory function, we performed high resolution respirometry on freshly isolated kidney tissue [40] (Fig. 5D). Under basal conditions, respiration rates were comparable between aged WT and sEH null kidneys, where tissues where reliant on endogenous respiratory demand (Figure S3A). However, after the addition of saturating concentrations of glutamate and malate to induce complex I-driven ATP synthesis respiration, aged sEH null kidneys showed significantly enhanced respiratory activity compared to the WT mice (Figure S3B). Enhanced mitochondrial performance in aged sEH null mice was further supported by higher respiratory control ratio (RCR), suggesting tighter coupling between the electron transport chain and ATP synthesis (Fig. 5E), increased coupling efficiency ratio, and elevated maximal mitochondrial respiratory capacity (Fig. 5F–G), indicating greater resilience to metabolic stress. To distinguish between changes in mitochondrial quality versus quantity, respiratory data was normalized to citrate synthase activity. After normalization, differences between groups were no longer evident (Fig. 5H–J and S3C,D), suggesting that the observed improvements in respiration in sEH null mice were due to increased mitochondrial abundance rather than enhanced quality.

## Discussion

Aging is a complex process characterized by gradual loss of physiological function leading to increased vulnerability to stress and disease [10]. Individuals over the age of 70 exhibit a 13% higher incidence of kidney disease compared to younger people [57]. Understanding the regulatory pathways that drive kidney aging will provide insight into the development of strategies to mitigate age-related renal dysfunction. In this study, we demonstrated that age-related changes in the kidney are marked by disrupted mitochondrial homeostasis, activation of senescence pathways, and elevated expression of SASP components, which coincided with increased expression of soluble epoxide hydrolase in aged kidneys. Notably, genetic deletion of sEH preserved mitochondrial content and dynamics, limited mitochondrial damage, and attenuated cellular senescence, collectively supporting a healthier renal environment in aged mice.

Previous studies suggest sEH has a role in the pathology observed across multiple organs such as the brain [20], heart [18, 19], intestines [21], liver [22], and kidney [18] in both human and murine models. For example, elevated sEH expression has been observed in heart tissue from patients with ischemic cardiomyopathy compared to non-failing controls [58]. In experimental models, pharmacological inhibition of sEH has shown protective effects in diabetic nephropathy, significantly reducing tubular injury in the kidney [28]. Moreover, epoxyeicosatrienoic acid (EETs), an epoxylipid rapidly hydrolyzed by sEH, has been demonstrated to suppress inflammation and reduce kidney fibrosis in animal models of chronic kidney diseases [14–16]. The current data confirms an age-related renal decline, exemplified by increased levels of the kidney injury marker, *Kim-1*, and elevated plasma creatinine and urea nitrogen levels, which were attenuated in sEH null mice. Histological images indicate aged mice show features of acute tubular and glomerular injury, which was more prominent in the WT than sEH null mice. Several studies have investigated the role of kidney-specific sEH deletion in renal injury. For example, targeted disruption of sEH in podocyte and glomerular epithelial cells has been shown to protect against renal damage by preserving EET levels and attenuating NF-κB signaling activation [26, 59]. In contrast, in vitro studies using rat proximal tubular cells demonstrated that upregulation of sEH exacerbates cellular injury [60]. Similarly, Liu et al. [61] reported that oral administration of sEH inhibitor AR9273 (1-adamantan-1-yl-3-(1-methylsulfonyl-piperidin-4-yl-urea) mitigated cisplatin-induced nephrotoxicity by reducing tubular damage [61]. Collectively, these findings highlight the pathological relevance of elevated sEH expression in kidney disease and underscore its role in disrupting the balance of bioactive lipid mediators. Specifically, increased sEH activity accelerates the hydrolysis of N-3 and N-6 epoxylipids into their diol metabolites, diminishing their protective effects. Consequently, sEH deletion may exert intrinsic renal protective effects, complementing the systemic benefits observed in aged sEH null mice.

Despite the clinical significance of aging, older patients are often excluded from clinical trials, limiting our understanding of contributing factors that mediate these differences in the outcomes [62, 63]. The current study utilized 24-month-old mice to better reflect changes observed in an individual approximately 65 to 70 years old [30]. Assessment of a frailty index was employed to measure aging-related health changes and provide an indication of overall physiological decline [34, 37, 64–66]. Aged mice normally exhibit significantly higher frailty index scores than younger animals, correlating with structural and functional impairments [67]. The lower frailty index observed in sEH null mice is consistent with previously reported data demonstrating aged sEH null females have a significantly prolonged lifespan compared to WT [23]. Moreover, aged sEH null mice demonstrate better survivability and disease tolerability in models of acute myocardial injury or endotoxemia [19, 23, 33]. Growing evidence suggests the protective effects of sEH inhibition involve preserving mitochondrial health, which can limit cellular senescence in aged mice [19, 21, 68–72]. Cellular senescence, a hallmark of aging, is characterized by the activation of the p53/p21 and p16INK4a pathways. Induction of senescence markers such as *p16*,* p21*, and *p53* can reflect cellular senescence in both kidney injury and aging models. For example, a study of human kidneys found a strong correlation between *p16INK4A* expression and age-related histopathological features, including glomerulosclerosis, interstitial fibrosis, and tubular atrophy, supporting a direct renal cell senescence and organ aging [42, 73]. Similarly, in patients with IgA nephropathy, elevated *p16* and *p21* expression in renal tubular cells is associated with disease progression, independent of systemic factors such as chronological age or obesity [74]. In a murine model, a high-fat diet induced renal tubular senescence, marked by upregulation of *p16*,* p19*, and *p53*, emphasizing a local, rather than systemic, origin of senescence in renal tissue [75]. The elevated expression of senescence markers observed in aged WT kidneys is consistent with the accumulation of age-related deficits in physiological, functional, and behavioral parameters identified with the frailty index. This is aligns with prior studies showing *p16*-positive cells accumulate in the kidneys of aged mice [76, 77], and that the number of *p16*- and *p21*-cells increases with age in human kidneys [42, 78]. Increased *p21* and *p16* contributes to cell cycle arrest and promotes the secretion of SASP factors, thereby driving chronic inflammation and tissue deterioration characterized in aging [79]. Several studies support the idea of deleting or attenuating senescence may help reduce age-related functional changes. For example, deletion of *p16* attenuated kidney senescence and ameliorated injury [42, 80]. Similarly, the removal of *p21* improved the renal function in age related diseases [81, 82]. In the present study, we observed a significant increase in p21⁺ cells in aged kidneys (24 months old), associated with reduced renal function. This aligns with previous studies showing age-dependent increases in p21⁺ cells became evident by 18 months, along with structural and histological changes [6, 83, 84]. This likely reflects the accumulation of DNA damage in renal cells, promoting a senescent phenotype and upregulation of senescence markers with advanced age [6, 83, 84]. Importantly, sEH null mice did not exhibit age-related increase in senescent markers, *p21*,* p53*, and *p16*, observed in WT mice, suggesting a potential link between the increased sEH expression and the promotion of renal senescence with aging.

The progressive buildup of senescent cells is a key driver of aging by altering local physiological functions through autocrine and paracrine SASP signaling. Senescent cells undergo disruptions in cellular homeostasis, marked by the activation of and release of pro-inflammatory mediators—including cytokines, chemokines, proteases, and growth factors—that contribute to a state of chronic inflammation, often referred to as “inflammaging” [43]. Sustained SASP expression creates a chronically pro-inflammatory microenvironment that accelerates tissue dysfunction and the development of age-related conditions [44]. In the current study, we present key components of the SASP and the primary regulatory pathways activated in senescent cells, including IL-1β, caspase-1, MCP-1, CD68, and interferons, which are closely linked to heightened chronic inflammation and the progression of aging-related conditions [44, 85]. Notably, sEH deletion can modulate SASP expression, highlighting its potential role in controlling age-related inflammatory signaling [19, 69].

Aging is a multifaceted process resulting from the cumulative effects of cellular damage, metabolic dysregulation, epigenetic modifications, and systemic functional decline in organ function. Caveolin-1, a structural protein of caveolae, has emerged as an important regulator of signaling pathways involved in cellular senescence, oxidative stress, and inflammation [86, 87]. Correlational evidence demonstrates increased Cav-1 expression in aged human diploid fibroblasts, senescent mesenchymal stem cells, and bone marrow stromal cells [88–91]. Aligning with these observations, elevated caveolin-1 levels are found in the lungs, spleen, skeletal muscle, and brain of aged rodents [92, 93]. Accumulation of senescent cells leads to the release of growth factors and inflammatory mediators having paracrine effects; Cav-1 plays a central role in modulating the signal transduction thus enhancing the senescent phenotypes [86]. Growth factors and oxidative stress are extracellular stimuli known to regulate Cav1 phosphorylation, for example, induction of oxidative stress by endotoxin LPS, and cytokine mixture (IL-1ß and TNF-α) regulate Cav1 phosphorylation in mouse lung endothelial cells [94, 95]. Importantly, phosphorylation of Cav-1 at tyrosine 14 is a key post-translational modification that alters its function in cellular signaling and senescence. Evidence has demonstrated phosphorylated Cav-1 is associated with activation of p53/p21 pathways and contributes to SASP [96–98]. In this study, we observed a significant increase in phosphorylated Cav-1 in both plasma membrane and mitochondria fractions of aged WT kidneys, correlating with enhanced activation of cellular senescence pathways. The attenuation of this response following sEH deletion suggests a potential senotherapeutic effect involving modulating Cav-1 signaling and downstream events.

The main factors contributing to cellular senescence and halt the cell cycle include oxidative stress, DNA mutations, and mitochondrial mediated events [79, 99]. Mitochondria are highly dynamic organelles, constantly altering their number and size in response to energy demands. They undergo continuous fusion and fission processes, switching between elongated interconnected networks and fragmented discrete structures. These dynamics are crucial for maintaining mitochondrial energy production, function, and overall cellular homeostasis [100]. However, mitochondrial theory of aging suggests that aging disrupts mitochondrial bioenergetic capacity and dynamics leading to dysfunction [101–103]. A decline in mitochondrial bioenergetic capacity can result from reduced mitochondrial biogenesis, impaired mitochondrial dynamics, increased mitophagy, diminished energy production, or a combination of those processes [104]. In the present study, aged WT mice exhibited a reduction in mtDNA copy number and citrate synthase activity, indicative of age-related loss of mitochondrial content. Notably, these declines were attenuated in aged sEH null kidneys, suggesting a protective role of sEH deletion in preserving mitochondrial mass during aging.

Clinical studies have associated reduced mitochondrial DNA copy number with adverse outcomes, likely reflecting impaired mitochondrial function [105, 106]. Compared to aged WT mice, aged sEH null kidneys displayed greater mitochondrial content and a higher respiratory ratio (RCR), suggesting preserved mitochondrial content with aging and more efficient coupling of oxygen consumption to ATP synthesis [107]. However, when respiratory parameters were normalized to citrate synthase activity, as proxy for mitochondrial mass, the differences between aged WT and sEH null were no longer apparent. This suggests that the preserved respiratory capacity observed in aged sEH null kidneys is attributable to a higher mitochondrial abundance, rather than intrinsic improvements in mitochondrial function. Interestingly, in addition to reduced mitochondrial content, our findings reveal that aged WT mice exhibited evidence of significant accumulation of damaged mitochondria reflected by elevated plasma GDF-15 levels, an established marker of mitochondrial stress, which was absent in sEH-null mice [108]. However, the precise tissue or cellular source of GDF-15 production remains unclear. Further investigation into age-related changes into renal mitochondria included assessment of the release of mtDNA into the cytosol, a process known to activate innate immune responses with the pattern recognition receptors (PRRs), including toll-like receptor 9 (TLR9), cyclic GMP-AMP synthase (cGAS), and Z-DNA binding protein 1 (zBP1) [109–112]. Our results showed that aged WT kidneys had increased cytosolic mtDNA, accompanied by upregulation of *zBP1*, a cytosolic sensor of nucleic acids known to activate interferon responses. This was associated with increased expression of *Ifit-1* and *Ifnb1*, two classical interferon-stimulated genes. Together, these findings suggest that mitochondrial damage in aged WT kidneys may promote chronic inflammation through zBP1-mediated interferon signaling, potentially contributing to renal aging [113].

**Fig. 6:**
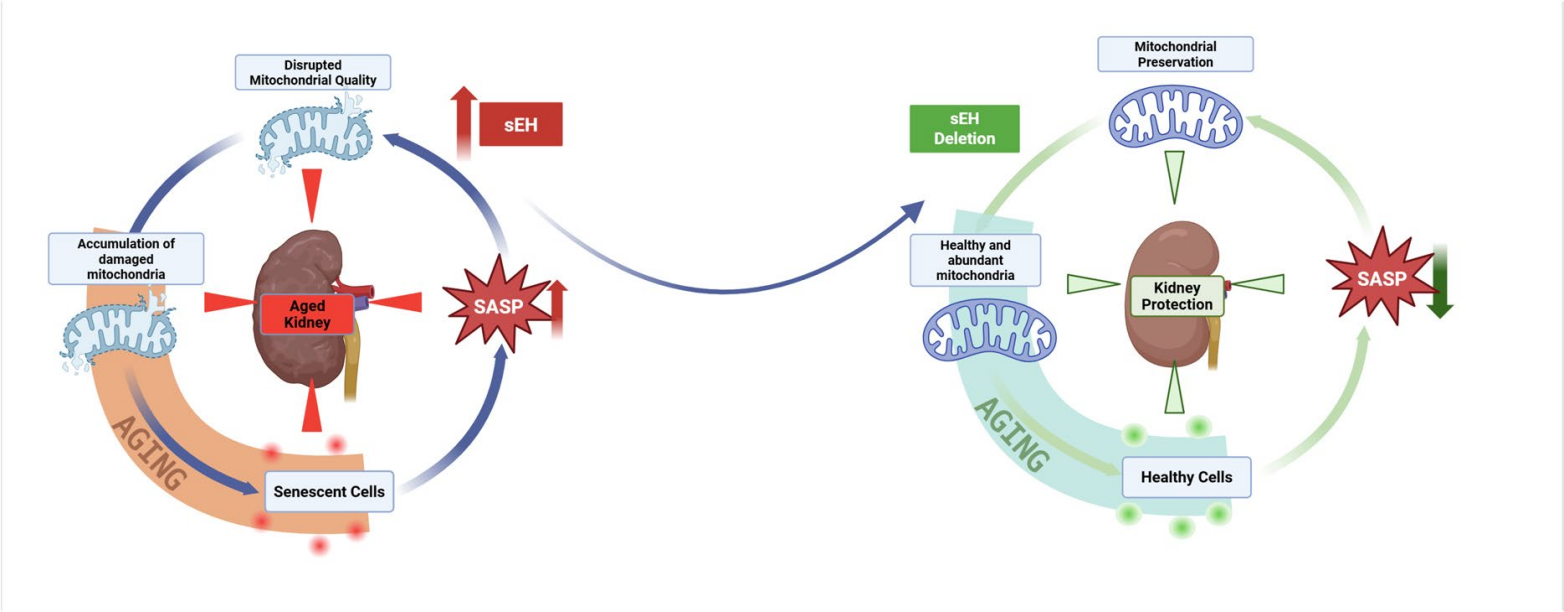
Schematic presentation illustrating the beneficial effects of sEH deletion on the aging process in female kidneys. Aging is associated with increased sEH levels and a decline in kidney function, characterized by impaired mitochondrial biology, reduced mitochondrial respiration, elevated senescence markers, and increased inflammatory and SASP markers

Our current data demonstrating sEH deletion suppressed the age-induced mitochondrial imbalance, aligning with previous studies showing that sEH deficiency reduces tissue damage by preserving mitochondrial quality, content, and respiration, while attenuating cellular senescence and SASP expression [19, 23, 54, 68, 70, 71]. Collectively, these results support the notion that epoxide hydrolases contribute to the renal aging. Importantly, deletion of sEH preserved mitochondrial integrity, limited senescence, and mitigated the detrimental effects of aging on kidney function, suggesting a healthy renal environment in aged mice (Fig. 6). This study has several limitations. First, the use of a whole-body sEH knockout model limits our ability to distinguishing between kidney-specific and systemic effects, complicating the interpretation of whether the observed renal benefits are directly attributable to local sEH deletion. Second, our focus on female mice, while biologically justified, may restrict the generalizability of our findings. Female mice display distinct age-related changes in mitochondrial function, inflammation, and cardiovascular risk profiles that are not consistently replicated in males [18]. Third, our immunofluorescence images show nuclear and perinuclear p21 expression, which may be influenced by staining conditions; however, subcellular variation in p21 localization has also been reported by others [114–116]. Finally, while our data demonstrates important phenotypic differences, many of the measurements are descriptive rather than mechanistic, highlighting the need for future studies to explore the underlying molecular pathways in more detail.

## Conclusion

Aging is associated with significant physiological changes, notably in the kidneys, resulting in reduced function and heightened vulnerability to injury and disease. Our study highlights a role for sEH in age-related kidney dysfunction, as its deletion was shown to maintain mitochondrial homeostasis and attenuating senescence markers, ultimately preserving kidney health. Together, these data suggest targeting sEH may offer a promising strategy to mitigate adverse effects of aging by preserving mitochondrial quality and reducing cellular senescence.

## Supplementary Information

Below is the link to the electronic supplementary material.

Supplementary File 1 (DOCX 889 KB)

## Data Availability

Data will be made available upon reasonable request.

## References

[1] Liu P, Quinn RR, Lam NN, Elliott MJ, Xu Y, James MT, et al. Accounting for age in the definition of chronic kidney disease. JAMA Intern Med. 2021;181(10):1359–66. 34459844 10.1001/jamainternmed.2021.4813 PMC8406213

[2] North BJ, Sinclair DA. The intersection between aging and cardiovascular disease. Circ Res. 2012;110(8):1097–108. 22499900 10.1161/CIRCRESAHA.111.246876 PMC3366686

[3] Zhou XJ, Rakheja D, Yu X, Saxena R, Vaziri ND, Silva FG. The aging kidney. Kidney Int. 2008;74(6):710–20. 18614996 10.1038/ki.2008.319

[4] Fang Y, Gong AY, Haller ST, Dworkin LD, Liu Z, Gong R. The ageing kidney: molecular mechanisms and clinical implications. Ageing Res Rev. 2020;63101151. 10.1016/j.arr.2020.101151 PMC7595250 32835891

[5] Yamamoto T, Isaka Y. Pathological mechanisms of kidney disease in ageing. Nat Rev Nephrol. 2024;1–13. 10.1038/s41581-024-00868-4 39025993

[6] Marquez-Exposito L, Tejedor-Santamaria L, Valentijn FA, Tejera-Muñoz A, Rayego-Mateos S, Marchant V, et al. Oxidative stress and cellular senescence are involved in the aging kidney. Antioxidants. 2022;11(2):301. 35204184 10.3390/antiox11020301 PMC8868560

[7] Coppe JP, Patil CK, Rodier F, Sun Y, Munoz DP, Goldstein J, et al. Senescence-associated secretory phenotypes reveal cell-nonautonomous functions of oncogenic RAS and the p53 tumor suppressor. PLoS Biol. 2008;6(12):2853–68. 10.1371/journal.pbio.0060301. 19053174 10.1371/journal.pbio.0060301 PMC2592359

[8] Docherty M-H, O’Sullivan ED, Bonventre JV, Ferenbach DA. Cellular senescence in the kidney. J Am Soc Nephrol. 2019;30(5):726–36. 31000567 10.1681/ASN.2018121251 PMC6493983

[9] Lopez-Otin C, Blasco MA, Partridge L, Serrano M, Kroemer G. The hallmarks of aging. Cell. 2013;153(6):1194–217. 10.1016/j.cell.2013.05.039. 23746838 10.1016/j.cell.2013.05.039 PMC3836174

[10] Lopez-Otin C, Blasco MA, Partridge L, Serrano M, Kroemer G. Hallmarks of aging: an expanding universe. Cell. 2023;186(2):243–78. 10.1016/j.cell.2022.11.001. 36599349 10.1016/j.cell.2022.11.001

[11] Andrieux P, Chevillard C, Cunha-Neto E, Nunes JPS. Mitochondria as a cellular hub in infection and inflammation. Int J Mol Sci. 2021;22(21):11338. 34768767 10.3390/ijms222111338 PMC8583510

[12] Wiley CD, Velarde MC, Lecot P, Liu S, Sarnoski EA, Freund A, et al. Mitochondrial dysfunction induces senescence with a distinct secretory phenotype. Cell Metab. 2016;23(2):303–14. 10.1016/j.cmet.2015.11.011. 26686024 10.1016/j.cmet.2015.11.011 PMC4749409

[13] Fontecha-Barriuso M, Lopez-Diaz AM, Guerrero-Mauvecin J, Miguel V, Ramos AM, Sanchez-Niño MD, et al. Tubular mitochondrial dysfunction, oxidative stress, and progression of chronic kidney disease. Antioxidants. 2022;11(7):1356. 35883847 10.3390/antiox11071356 PMC9311633

[14] Imig JD. Prospective for cytochrome P450 epoxygenase cardiovascular and renal therapeutics. Pharmacol Ther. 2018;1921–19. 10.1016/j.pharmthera.2018.06.015. 10.1016/j.pharmthera.2018.06.015 PMC6263841 29964123

[15] Kim J, Imig JD, Yang J, Hammock BD, Padanilam BJ. Inhibition of soluble epoxide hydrolase prevents renal interstitial fibrosis and inflammation. Am J Physiol Renal Physiol. 2014;307(8):F971–80. 10.1152/ajprenal.00256.2014. 25164080 10.1152/ajprenal.00256.2014 PMC4200297

[16] Kim J, Yoon SP, Toews ML, Imig JD, Hwang SH, Hammock BD, et al. Pharmacological inhibition of soluble epoxide hydrolase prevents renal interstitial fibrogenesis in obstructive nephropathy. Am J Physiol Renal Physiol. 2015;308(2):F131–9. 10.1152/ajprenal.00531.2014. 25377915 10.1152/ajprenal.00531.2014 PMC4338924

[17] Morisseau C, Hammock BD. Impact of soluble epoxide hydrolase and epoxyeicosanoids on human health. Annu Rev Pharmacol Toxicol. 2013;5337–58. 10.1146/annurev-pharmtox-011112-140244. 10.1146/annurev-pharmtox-011112-140244 PMC3578707 23020295

[18] Jamieson KL, Keshavarz-Bahaghighat H, Darwesh AM, Sosnowski DK, Seubert JM. Age and sex differences in hearts of soluble epoxide hydrolase null mice. Front Physiol. 2020;1148. 10.3389/fphys.2020.00048. 10.3389/fphys.2020.00048 PMC7019103 32116760

[19] Yousef A, Sosnowski DK, Fang L, Legaspi RJ, Korodimas J, Lee A, et al. Cardioprotective response and senescence in aged sEH null female mice exposed to LPS. Am J Physiol Heart Circ Physiol. 2024;326(6):H1366–85. 10.1152/ajpheart.00706.2023. 38578240 10.1152/ajpheart.00706.2023

[20] Nelson JW, Young JM, Borkar RN, Woltjer RL, Quinn JF, Silbert LC, et al. Role of soluble epoxide hydrolase in age-related vascular cognitive decline. Prostaglandins Other Lipid Mediat. 2014;113–11530–7. 10.1016/j.prostaglandins.2014.09.003. 10.1016/j.prostaglandins.2014.09.003 PMC4254026 25277097

[21] Wang W, Wagner KM, Wang Y, Singh N, Yang J, He Q, et al. Soluble epoxide hydrolase contributes to cell senescence and ER stress in aging mice colon. Int J Mol Sci. 2023;24(5). 10.3390/ijms24054570. 10.3390/ijms24054570 PMC10003560 36901999

[22] Wu Y, Dong J-H, Dai Y-F, Zhu M-Z, Wang M-Y, Zhang Y, et al. Hepatic soluble epoxide hydrolase activity regulates cerebral Aβ metabolism and the pathogenesis of Alzheimer’s disease in mice. 2023. 10.1016/j.neuron.2023.06.002 37402372

[23] Jamieson KL, Darwesh AM, Sosnowski DK, Zhang H, Shah S, Zhabyeyev P, et al. Soluble epoxide hydrolase in aged female mice and human explanted hearts following ischemic injury. Int J Mol Sci. 2021;22(4). 10.3390/ijms22041691. 10.3390/ijms22041691 PMC7915306 33567578

[24] McReynolds C, Morisseau C, Wagner K, Hammock B. Epoxy fatty acids are promising targets for treatment of pain, cardiovascular disease and other indications characterized by mitochondrial dysfunction, endoplasmic stress and inflammation. Adv Exp Med Biol. 2020;127471–99. 10.1007/978-3-030-50621-6_5. 10.1007/978-3-030-50621-6_5 PMC7737916 32894508

[25] Zuloaga KL, Zhang W, Roese NE, Alkayed NJJFiP. Soluble epoxide hydrolase gene deletion improves blood flow and reduces infarct size after cerebral ischemia in reproductively senescent female mice. 2015;5290. 10.3389/fphar.2014.00290 PMC4295540 25642188

[26] Bettaieb A, Koike S, Chahed S, Zhao Y, Bachaalany S, Hashoush N, et al. Podocyte-specific soluble epoxide hydrolase deficiency in mice attenuates acute kidney injury. FEBS J. 2017;284(13):1970–86. 10.1111/febs.14100. 28485854 10.1111/febs.14100 PMC5515292

[27] Loch D, Hoey A, Morisseau C, Hammock BO, Brown L. Prevention of hypertension in DOCA-salt rats by an inhibitor of soluble epoxide hydrolase. Cell Biochem Biophys. 2007;47(1):87–98. 10.1385/cbb:47:1:87. 17406062 10.1385/cbb:47:1:87 PMC1892223

[28] Jiang XS, Xiang XY, Chen XM, He JL, Liu T, Gan H, et al. Inhibition of soluble epoxide hydrolase attenuates renal tubular mitochondrial dysfunction and ER stress by restoring autophagic flux in diabetic nephropathy. Cell Death Dis. 2020;11(5):385. 10.1038/s41419-020-2594-x. 10.1038/s41419-020-2594-x PMC7242354 32439839

[29] Elmarakby AA, Faulkner J, Al-Shabrawey M, Wang MH, Maddipati KR, Imig JD. Deletion of soluble epoxide hydrolase gene improves renal endothelial function and reduces renal inflammation and injury in streptozotocin-induced type 1 diabetes. Am J Physiol Regul Integr Comp Physiol. 2011;301(5):R1307–17. 10.1152/ajpregu.00759.2010. 21832210 10.1152/ajpregu.00759.2010 PMC3213948

[30] Flurkey KM, Currer J, Harrison DE. Chapter 20 - Mouse models in aging research. In: Fox JG, Davisson MT, Quimby FW, Barthold SW, Newcomer CE, Smith AL, editors. The mouse in biomedical research. 2nd ed. Burlington: Academic Press; 2007. pp. 637–72.

[31] Sinal CJ, Miyata M, Tohkin M, Nagata K, Bend JR, Gonzalez FJ. Targeted disruption of soluble epoxide hydrolase reveals a role in blood pressure regulation. J Biol Chem. 2000;275(51):40504–10. 10.1074/jbc.M008106200. 11001943 10.1074/jbc.M008106200

[32] Olfert ED, Cross BM, McWilliam AA. Guide to the care and use of experimental animals: Citeseer. 1993.

[33] Sosnowski DK, Jamieson KL, Gruzdev A, Li Y, Valencia R, Yousef A, et al. Cardiomyocyte-specific disruption of soluble epoxide hydrolase limits inflammation to preserve cardiac function. Am J Physiol Heart Circ Physiol. 2022;323(4):H670–87. 10.1152/ajpheart.00217.2022. 35985007 10.1152/ajpheart.00217.2022 PMC9512117

[34] Whitehead JC, Hildebrand BA, Sun M, Rockwood MR, Rose RA, Rockwood K, et al. A clinical frailty index in aging mice: comparisons with frailty index data in humans. J Gerontol A Biol Sci Med Sci. 2014;69(6):621–32. 10.1093/gerona/glt136. 24051346 10.1093/gerona/glt136 PMC4022099

[35] Baines CP, Zhang J, Wang GW, Zheng YT, Xiu JX, Cardwell EM, et al. Mitochondrial PKCepsilon and MAPK form signaling modules in the murine heart: enhanced mitochondrial PKCepsilon-MAPK interactions and differential MAPK activation in PKCepsilon-induced cardioprotection. Circ Res. 2002;90(4):390–7. 10.1161/01.res.0000012702.90501.8d. 11884367 10.1161/01.res.0000012702.90501.8d

[36] Kranrod JW, Darwesh AM, Bassiouni W, Huang A, Fang L, Korodimas JV, et al. Cardioprotective action of a novel synthetic 19,20-EDP analog is SIRT dependent. J Cardiovasc Pharmacol. 2024;83(1):105–15. 10.1097/FJC.0000000000001495. 38180457 10.1097/FJC.0000000000001495 PMC10770468

[37] Livak KJ, Schmittgen TD. Analysis of relative gene expression data using real-time quantitative PCR and the 2(-delta delta C(T)) method. Methods. 2001;25(4):402–8. 10.1006/meth.2001.1262. 11846609 10.1006/meth.2001.1262

[38] Quiros PM, Goyal A, Jha P, Auwerx J. Analysis of mtDNA/nDNA ratio in mice. Curr Protoc Mouse Biol. 2017;7(1):47–54. 10.1002/cpmo.21. 28252199 10.1002/cpmo.21 PMC5335900

[39] Spinazzi M, Casarin A, Pertegato V, Salviati L, Angelini C. Assessment of mitochondrial respiratory chain enzymatic activities on tissues and cultured cells. Nat Protoc. 2012;7(6):1235–46. 10.1038/nprot.2012.058. 22653162 10.1038/nprot.2012.058

[40] Valencia R, Kranrod JW, Fang L, Soliman AM, Azer B, Clemente-Casares X, et al. Linoleic acid-derived diol 12,13-DiHOME enhances NLRP3 inflammasome activation in macrophages. FASEB J. 2024;38(13): e23748. 10.1096/fj.202301640RR.38940767 10.1096/fj.202301640RR

[41] Zhabyeyev P, McLean B, Bassiouni W, Valencia R, Paul M, Darwesh AM, et al. Loss of PI3Kalpha mediates protection from myocardial ischemia-reperfusion injury linked to preserved mitochondrial function. J Am Heart Assoc. 2023;12(12): e022352. 10.1161/JAHA.122.022352. 37318009 10.1161/JAHA.122.022352 PMC10356046

[42] Melk A, Schmidt BM, Takeuchi O, Sawitzki B, Rayner DC, Halloran PF. Expression of p16INK4a and other cell cycle regulator and senescence associated genes in aging human kidney. Kidney Int. 2004;65(2):510–20. 10.1111/j.1523-1755.2004.00438.x. 14717921 10.1111/j.1523-1755.2004.00438.x

[43] Lopes-Paciencia S, Saint-Germain E, Rowell M-C, Ruiz AF, Kalegari P, Ferbeyre GJC. The senescence-associated secretory phenotype and its regulation. 2019;11715–22. 10.1016/j.cyto.2019.01.013 30776684

[44] Wang WJ, Cai GY, Chen XM. Cellular senescence, senescence-associated secretory phenotype, and chronic kidney disease. Oncotarget. 2017;8(38):64520–33. 10.18632/oncotarget.17327. 10.18632/oncotarget.17327 PMC5610023 28969091

[45] Yu DM, Jung SH, An HT, Lee S, Hong J, Park JS, et al. Caveolin-1 deficiency induces premature senescence with mitochondrial dysfunction. Aging Cell. 2017;16(4):773–84. 10.1111/acel.12606. 28514055 10.1111/acel.12606 PMC5506423

[46] Feng X, Gao W, Li Y. Caveolin-1 is involved in high glucose accelerated human glomerular mesangial cell senescence. Korean J Intern Med. 2017;32(5):883–9. 10.3904/kjim.2015.254. 27048255 10.3904/kjim.2015.254 PMC5583444

[47] Jiang Y, Krantz S, Qin X, Li S, Gunasekara H, Kim YM, et al. Caveolin-1 controls mitochondrial damage and ROS production by regulating fission - fusion dynamics and mitophagy. Redox Biol. 2022;52102304. 10.1016/j.redox.2022.102304. 10.1016/j.redox.2022.102304 PMC9018165 35413643

[48] Ago T, Sadoshima J. GDF15, a cardioprotective TGF-beta superfamily protein. Circ Res. 2006;98(3):294–7. 10.1161/01.RES.0000207919.83894.9d. 16484622 10.1161/01.RES.0000207919.83894.9d

[49] Rochette L, Dogon G, Zeller M, Cottin Y, Vergely C. GDF15 and cardiac cells: current concepts and new insights. Int J Mol Sci. 2021;22(16). 10.3390/ijms22168889. 10.3390/ijms22168889 PMC8396208 34445593

[50] Jiang J, Wen W, Sachdev PS. Macrophage inhibitory cytokine-1/growth differentiation factor 15 as a marker of cognitive ageing and dementia. Curr Opin Psychiatry. 2016;29(2):181–6. 10.1097/YCO.0000000000000225. 26731555 10.1097/YCO.0000000000000225

[51] Srivastava A, Tomar B, Sharma D, Rath SK. Mitochondrial dysfunction and oxidative stress: role in chronic kidney disease. Life Sci. 2023;319121432. 10.1016/j.lfs.2023.121432. 10.1016/j.lfs.2023.121432 36706833

[52] Jamieson KL, Endo T, Darwesh AM, Samokhvalov V, Seubert JM. Cytochrome P450-derived eicosanoids and heart function. Pharmacol Ther. 2017;17947–83. 10.1016/j.pharmthera.2017.05.005. 10.1016/j.pharmthera.2017.05.005 28551025

[53] Seubert J, Yang B, Bradbury JA, Graves J, Degraff LM, Gabel S, et al. Enhanced postischemic functional recovery in CYP2J2 transgenic hearts involves mitochondrial ATP-sensitive K+ channels and p42/p44 MAPK pathway. Circ Res. 2004;95(5):506–14. 10.1161/01.RES.0000139436.89654.c8. 15256482 10.1161/01.RES.0000139436.89654.c8

[54] Seubert JM, Sinal CJ, Graves J, DeGraff LM, Bradbury JA, Lee CR, et al. Role of soluble epoxide hydrolase in postischemic recovery of heart contractile function. Circ Res. 2006;99(4):442–50. 10.1161/01.RES.0000237390.92932.37. 16857962 10.1161/01.RES.0000237390.92932.37 PMC2072806

[55] Schunck WH, Konkel A, Fischer R, Weylandt KH. Therapeutic potential of omega-3 fatty acid-derived epoxyeicosanoids in cardiovascular and inflammatory diseases. Pharmacol Ther. 2018;183177–204. 10.1016/j.pharmthera.2017.10.016. 10.1016/j.pharmthera.2017.10.016 29080699

[56] Yousef A, Fang L, Heidari M, Kranrod J, Seubert JM. The role of CYP-sEH derived lipid mediators in regulating mitochondrial biology and cellular senescence: implications for the aging heart. Front Pharmacol. 2024;151486717. 10.3389/fphar.2024.1486717. 10.3389/fphar.2024.1486717 PMC11655241 39703395

[57] Minutolo R, Borrelli S, De Nicola L. CKD in the elderly: kidney senescence or blood pressure-related nephropathy? Am J Kidney Dis. 2015;66(2):184–6. 10.1053/j.ajkd.2015.05.004. 26210723 10.1053/j.ajkd.2015.05.004

[58] Sosnowski DK, Jamieson KL, Darwesh AM, Zhang H, Keshavarz-Bahaghighat H, Valencia R, et al. Changes in the left ventricular eicosanoid profile in human dilated cardiomyopathy. Front Cardiovasc Med. 2022;9879209. 10.3389/fcvm.2022.879209. 10.3389/fcvm.2022.879209 PMC9160304 35665247

[59] Bettaieb A, Koike S, Hsu MF, Ito Y, Chahed S, Bachaalany S, et al. Soluble epoxide hydrolase in podocytes is a significant contributor to renal function under hyperglycemia. Biochim Biophys Acta Gen Subj. 2017;1861(11 Pt A):2758–65. 10.1016/j.bbagen.2017.07.021. 10.1016/j.bbagen.2017.07.021 PMC5873293 28757338

[60] Wang Q, Pang W, Cui Z, Shi J, Liu Y, Liu B, et al. Upregulation of soluble epoxide hydrolase in proximal tubular cells mediated proteinuria-induced renal damage. Am J Physiol Renal Physiol. 2013;304(2):F168–76. 10.1152/ajprenal.00129.2012. 23152298 10.1152/ajprenal.00129.2012 PMC3543623

[61] Liu Y, Lu X, Nguyen S, Olson JL, Webb HK, Kroetz DL. Epoxyeicosatrienoic acids prevent cisplatin-induced renal apoptosis through a p38 mitogen-activated protein kinase-regulated mitochondrial pathway. Mol Pharmacol. 2013;84(6):925–34. 10.1124/mol.113.088302. 24092818 10.1124/mol.113.088302 PMC3834146

[62] Boyd CM, Wolff JL, Giovannetti E, Reider L, Weiss C, Xue Q-l, et al. Healthcare task difficulty among older adults with multimorbidity. Med Care. 2014;52S118-S25. 10.1097/MLR.0b013e3182a977da PMC3937858 24561750

[63] Divo MJ, Martinez CH, Mannino DM. Ageing and the epidemiology of multimorbidity. Eur Respir J. 2014;44(4):1055–68. 25142482 10.1183/09031936.00059814 PMC4918092

[64] Clegg A, Young J, Iliffe S, Rikkert MO, Rockwood K. Frailty in elderly people. Lancet. 2013;381(9868):752–62. 10.1016/S0140-6736(12)62167-9. 23395245 10.1016/S0140-6736(12)62167-9 PMC4098658

[65] Fried LP, Tangen CM, Walston J, Newman AB, Hirsch C, Gottdiener J, et al. Frailty in older adults: evidence for a phenotype. J Gerontol A Biol Sci Med Sci. 2001;56(3):M146–56. 10.1093/gerona/56.3.m146. 11253156 10.1093/gerona/56.3.m146

[66] Liu H, Graber TG, Ferguson-Stegall L, Thompson LV. Clinically relevant frailty index for mice. J Gerontol A Biol Sci Med Sci. 2014;69(12):1485–91. 10.1093/gerona/glt188. 24336799 10.1093/gerona/glt188 PMC4271019

[67] Parks RJ, Fares E, Macdonald JK, Ernst MC, Sinal CJ, Rockwood K, et al. A procedure for creating a frailty index based on deficit accumulation in aging mice. J Gerontol A Biol Sci Med Sci. 2012;67(3):217–27. 10.1093/gerona/glr193. 22021390 10.1093/gerona/glr193

[68] Jamieson KL, Samokhvalov V, Akhnokh MK, Lee K, Cho WJ, Takawale A, et al. Genetic deletion of soluble epoxide hydrolase provides cardioprotective responses following myocardial infarction in aged mice. Prostaglandins & other lipid mediators. 2017;13247–58. 10.1016/j.prostaglandins.2017.01.001 28104457

[69] Keshavarz-Bahaghighat H, Darwesh AM, Sosnowski DK, Seubert JM. Mitochondrial dysfunction and inflammaging in heart failure: novel roles of CYP-derived epoxylipids. Cells. 2020;9(7). 10.3390/cells9071565. 10.3390/cells9071565 PMC7408578 32604981

[70] Jiang X-S, Xiang X-Y, Chen X-M, He J-L, Liu T, Gan H, et al. Inhibition of soluble epoxide hydrolase attenuates renal tubular mitochondrial dysfunction and ER stress by restoring autophagic flux in diabetic nephropathy. 2020;11(5):385. 10.1038/s41419-020-2594-x PMC7242354 32439839

[71] Darwesh AM, Keshavarz-Bahaghighat H, Jamieson KL, Seubert JM. Genetic deletion or pharmacological inhibition of soluble epoxide hydrolase ameliorates cardiac ischemia/reperfusion injury by attenuating NLRP3 inflammasome activation. Int J Mol Sci. 2019;20(14). 10.3390/ijms20143502. 10.3390/ijms20143502 PMC6678157 31319469

[72] Batchu SN, Lee SB, Samokhvalov V, Chaudhary KR, El-Sikhry H, Weldon SM, et al. Novel soluble epoxide hydrolase inhibitor protects mitochondrial function following stress. Can J Physiol Pharmacol. 2012;90(6):811–23. 10.1139/y2012-082. 22624559 10.1139/y2012-082

[73] Sis B, Tasanarong A, Khoshjou F, Dadras F, Solez K, Halloran PF. Accelerated expression of senescence associated cell cycle inhibitor p16INK4A in kidneys with glomerular disease. Kidney Int. 2007;71(3):218–26. 10.1038/sj.ki.5002039. 17183247 10.1038/sj.ki.5002039

[74] Liu J, Yang JR, He YN, Cai GY, Zhang JG, Lin LR, et al. Accelerated senescence of renal tubular epithelial cells is associated with disease progression of patients with immunoglobulin A (IgA) nephropathy. Transl Res. 2012;159(6):454–63. 10.1016/j.trsl.2011.11.008. 22633096 10.1016/j.trsl.2011.11.008

[75] Kim SR, Jiang K, Ogrodnik M, Chen X, Zhu XY, Lohmeier H, et al. Increased renal cellular senescence in murine high-fat diet: effect of the senolytic drug quercetin. Transl Res. 2019;213112–23. 10.1016/j.trsl.2019.07.005. 10.1016/j.trsl.2019.07.005 PMC6783353 31356770

[76] Berkenkamp B, Susnik N, Baisantry A, Kuznetsova I, Jacobi C, Sorensen-Zender I, et al. In vivo and in vitro analysis of age-associated changes and somatic cellular senescence in renal epithelial cells. PLoS ONE. 2014;9(2): e88071. 10.1371/journal.pone.0088071. 24505380 10.1371/journal.pone.0088071 PMC3913727

[77] Yousefzadeh MJ, Zhao J, Bukata C, Wade EA, McGowan SJ, Angelini LA, et al. Tissue specificity of senescent cell accumulation during physiologic and accelerated aging of mice. Aging Cell. 2020;19(3): e13094. 10.1111/acel.13094. 31981461 10.1111/acel.13094 PMC7059165

[78] Ueda S, Tominaga T, Ochi A, Sakurai A, Nishimura K, Shibata E, et al. TGF-β1 is involved in senescence-related pathways in glomerular endothelial cells via p16 translocation and p21 induction. Sci Rep. 2021;11(1):21643. 34737348 10.1038/s41598-021-01150-4 PMC8569175

[79] Kumari R, Jat PJFic, biology d. Mechanisms of cellular senescence: cell cycle arrest and senescence associated secretory phenotype. 2021;9645593. 10.3389/fcell.2021.645593 PMC8039141 33855023

[80] Baker DJ, Wijshake T, Tchkonia T, LeBrasseur NK, Childs BG, van de Sluis B, et al. Clearance of p16Ink4a-positive senescent cells delays ageing-associated disorders. Nature. 2011;479(7372):232–6. 10.1038/nature10600. 22048312 10.1038/nature10600 PMC3468323

[81] Megyesi J, Price PM, Tamayo E, Safirstein RL. The lack of a functional p21 WAF1/CIP1 gene ameliorates progression to chronic renal failure. Proc Natl Acad Sci. 1999;96(19):10830–5. 10485911 10.1073/pnas.96.19.10830 PMC17968

[82] Al-Dabet MdM, Shahzad K, Elwakiel A, Sulaj A, Kopf S, Bock F, et al. Reversal of the renal hyperglycemic memory in diabetic kidney disease by targeting sustained tubular p21 expression. Nat Commun. 2022;13(1):5062. 10.1038/s41467-022-32477-9 PMC9420151 36030260

[83] Johnson AC, Zager RA. Plasma and urinary p21: potential biomarkers of AKI and renal aging. Am J Physiol Renal Physiol. 2018;315(5):F1329–35. 10.1152/ajprenal.00328.2018. 30066587 10.1152/ajprenal.00328.2018 PMC6293288

[84] Kar P, Sivasailam A, Lavarti R, Cai L, Thangaraju M, Nguyen E, et al. p53 dependence of senescence markers p21v1 and p21v2 in aging and acute injury. NPJ Aging. 2024;10(1):45. 10.1038/s41514-024-00175-z. 39402059 10.1038/s41514-024-00175-z PMC11473800

[85] Li X, Li C, Zhang W, Wang Y, Qian P, Huang H. Inflammation and aging: signaling pathways and intervention therapies. Signal Transduct Target Ther. 2023;8(1):239. 10.1038/s41392-023-01502-8. 37291105 10.1038/s41392-023-01502-8 PMC10248351

[86] Volonte D, Galbiati F. Caveolin-1, a master regulator of cellular senescence. Cancer Metastasis Rev. 2020;39(2):397–414. 32279119 10.1007/s10555-020-09875-w PMC7890422

[87] Zou H, Stoppani E, Volonte D, Galbiati F. Caveolin-1, cellular senescence and age-related diseases. Mech Ageing Dev. 2011;132(11–12):533–42. 22100852 10.1016/j.mad.2011.11.001 PMC3243775

[88] Kang M-J, Chung YH, Hwang C-I, Murata M, Fujimoto T, Mook-Jung I-H, et al. Caveolin-1 upregulation in senescent neurons alters amyloid precursor protein processing. Exp Mol Med. 2006;38(2):126–33. 16672766 10.1038/emm.2006.16

[89] Bitar MS, Abdel-Halim SM, Al-Mulla F. Caveolin-1/PTRF upregulation constitutes a mechanism for mediating p53-induced cellular senescence: implications for evidence-based therapy of delayed wound healing in diabetes. American Journal of Physiology-Endocrinology and Metabolism. 2013;305(8):E951–63. 23941874 10.1152/ajpendo.00189.2013

[90] Park J-S, Kim H-Y, Kim H-W, Chae G-N, Oh H-T, Park J-Y, et al. Increased caveolin-1, a cause for the declined adipogenic potential of senescent human mesenchymal stem cells. Mech Ageing Dev. 2005;126(5):551–9. 15811424 10.1016/j.mad.2004.11.014

[91] Sun C, Wang N, Huang J, Xin J, Peng F, Ren Y, et al. Inhibition of phosphatidylcholine-specific phospholipase C prevents bone marrow stromal cell senescence in vitro. J Cell Biochem. 2009;108(2):519–28. 19626662 10.1002/jcb.22282

[92] Wicher SA, Prakash Y, Pabelick CM. Caveolae, caveolin-1 and lung diseases of aging. Expert Rev Respir Med. 2019;13(3):291–300. 30686114 10.1080/17476348.2019.1575733

[93] Park W-Y, Park J-S, Cho K-A, Kim D-I, Ko Y-G, Seo J-S, et al. Up-regulation of caveolin attenuates epidermal growth factor signaling in senescent cells. J Biol Chem. 2000;275(27):20847–52. 10781609 10.1074/jbc.M908162199

[94] Kim CA, Delépine M, Boutet E, El Mourabit H, Le Lay S, Meier M, et al. Association of a homozygous nonsense caveolin-1 mutation with Berardinelli-Seip congenital lipodystrophy. J Clin Endocrinol Metab. 2008;93(4):1129–34. 18211975 10.1210/jc.2007-1328

[95] Jiao H, Zhang Y, Yan Z, Wang Z-G, Liu G, Minshall RD, et al. Caveolin-1 Tyr14 phosphorylation induces interaction with TLR4 in endothelial cells and mediates MyD88-dependent signaling and sepsis-induced lung inflammation. J Immunol. 2013;191(12):6191–9. 24244013 10.4049/jimmunol.1300873 PMC3874812

[96] Goutas A, Outskouni Z, Papathanasiou I, Satra M, Koliakos G, Trachana V. Dysregulation of caveolin-1 phosphorylation and nuclear translocation is associated with senescence onset. Cells. 2021;10(11). 10.3390/cells10112939. 10.3390/cells10112939 PMC8616550 34831162

[97] Ha TY, Choi YR, Noh HR, Cha SH, Kim JB, Park SM. Age-related increase in caveolin-1 expression facilitates cell-to-cell transmission of alpha-synuclein in neurons. Mol Brain. 2021;14(1):122. 10.1186/s13041-021-00834-2. 34321069 10.1186/s13041-021-00834-2 PMC8320051

[98] Chretien A, Piront N, Delaive E, Demazy C, Ninane N, Toussaint O. Increased abundance of cytoplasmic and nuclear caveolin 1 in human diploid fibroblasts in H(2)O(2)-induced premature senescence and interplay with p38alpha(MAPK). FEBS Lett. 2008;582(12):1685–92. 10.1016/j.febslet.2008.04.026. 18439424 10.1016/j.febslet.2008.04.026

[99] Correia-Melo C, Marques FD, Anderson R, Hewitt G, Hewitt R, Cole J, et al. Mitochondria are required for pro-ageing features of the senescent phenotype. EMBO J. 2016;35(7):724–42. 10.15252/embj.201592862. 10.15252/embj.201592862 PMC4818766 26848154

[100] Tilokani L, Nagashima S, Paupe V, Prudent J. Mitochondrial dynamics: overview of molecular mechanisms. Essays Biochem. 2018;62(3):341–60. 10.1042/EBC20170104. 30030364 10.1042/EBC20170104 PMC6056715

[101] Seo AY, Joseph A-M, Dutta D, Hwang JCY, Aris JP, Leeuwenburgh C. New insights into the role of mitochondria in aging: mitochondrial dynamics and more. J Cell Sci. 2010;123(15):2533–42. 10.1242/jcs.070490. 20940129 10.1242/jcs.070490 PMC2912461

[102] Bereiter-Hahn J. Mitochondrial dynamics in aging and disease. Prog Mol Biol Transl Sci. 2014;12793–131. 10.1016/B978-0-12-394625-6.00004-0 25149215

[103] Harman D. The biologic clock: the mitochondria? J Am Geriatr Soc. 1972;20(4):145–7. 10.1111/j.1532-5415.1972.tb00787.x. 5016631 10.1111/j.1532-5415.1972.tb00787.x

[104] Guo Y, Guan T, Shafiq K, Yu Q, Jiao X, Na D, et al. Mitochondrial dysfunction in aging. Ageing Res Rev. 2023;88101955. 10.1016/j.arr.2023.101955 37196864

[105] Ashar FN, Zhang Y, Longchamps RJ, Lane J, Moes A, Grove ML, et al. Association of mitochondrial DNA copy number with cardiovascular disease. JAMA Cardiol. 2017;2(11):1247–55. 10.1001/jamacardio.2017.3683. 29049454 10.1001/jamacardio.2017.3683 PMC5710361

[106] Mengel-From J, Thinggaard M, Dalgard C, Kyvik KO, Christensen K, Christiansen L. Mitochondrial DNA copy number in peripheral blood cells declines with age and is associated with general health among elderly. Hum Genet. 2014;133(9):1149–59. 10.1007/s00439-014-1458-9. 24902542 10.1007/s00439-014-1458-9 PMC4127366

[107] Gnaiger E. Mitochondrial pathways and respiratory control: an introduction to OXPHOS analysis. Bioenergetics communications. 2020;20202.

[108] Montero R, Yubero D, Villarroya J, Henares D, Jou C, Rodriguez MA, et al. GDF-15 is elevated in children with mitochondrial diseases and is induced by mitochondrial dysfunction. PLoS ONE. 2016;11(2): e0148709. 10.1371/journal.pone.0148709. 26867126 10.1371/journal.pone.0148709 PMC4750949

[109] West AP, Shadel GS. Mitochondrial DNA in innate immune responses and inflammatory pathology. Nat Rev Immunol. 2017;17(6):363–75. 10.1038/nri.2017.21. 28393922 10.1038/nri.2017.21 PMC7289178

[110] Szczesny B, Marcatti M, Ahmad A, Montalbano M, Brunyanszki A, Bibli SI, et al. Mitochondrial DNA damage and subsequent activation of Z-DNA binding protein 1 links oxidative stress to inflammation in epithelial cells. Sci Rep. 2018;8(1):914. 10.1038/s41598-018-19216-1. 29343810 10.1038/s41598-018-19216-1 PMC5772643

[111] De Cecco M, Ito T, Petrashen AP, Elias AE, Skvir NJ, Criscione SW, et al. L1 drives IFN in senescent cells and promotes age-associated inflammation. Nature. 2019;566(7742):73–8. 10.1038/s41586-018-0784-9. 30728521 10.1038/s41586-018-0784-9 PMC6519963

[112] Riley JS, Tait SW. Mitochondrial DNA in inflammation and immunity. EMBO Rep. 2020;21(4):e49799. 10.15252/embr.201949799. 10.15252/embr.201949799 PMC7132203 32202065

[113] Lei Y, Guerra Martinez C, Torres-Odio S, Bell SL, Birdwell CE, Bryant JD, et al. Elevated type I interferon responses potentiate metabolic dysfunction, inflammation, and accelerated aging in mtDNA mutator mice. Sci Adv. 2021;7(22). 10.1126/sciadv.abe7548. 10.1126/sciadv.abe7548 PMC8153723 34039599

[114] Ghannam-Shahbari D, Jacob E, Kakun RR, Wasserman T, Korsensky L, Sternfeld O, et al. PAX8 activates a p53–p21-dependent pro-proliferative effect in high grade serous ovarian carcinoma. Oncogene. 2018;37(17):2213–24. 10.1038/s41388-017-0040-z. 29379162 10.1038/s41388-017-0040-z

[115] Maiuthed A, Ninsontia C, Erlenbach-Wuensch K, Ndreshkjana B, Muenzner JK, Caliskan A, et al. Cytoplasmic p21 mediates 5-fluorouracil resistance by inhibiting pro-apoptotic Chk2. Cancers (Basel). 2018;10(10). 10.3390/cancers10100373. 10.3390/cancers10100373 PMC6210175 30304835

[116] Lee J, Park S, Roh S. Y-27632, a ROCK inhibitor, delays senescence of putative murine salivary gland stem cells in culture. Arch Oral Biol. 2015;60(6):875–82. 10.1016/j.archoralbio.2015.03.003. 25804560 10.1016/j.archoralbio.2015.03.003

