# Supplementary material for: Alterations in mitochondria and cellular senescence in aged sEH null female kidneys
Source: GeroScience. 2025 Jul 26;48(2):2707–25. doi: 10.1007/s11357-025-01814-3 (PMC12972460; doi:10.1007/s11357-025-01814-3)
Supplement: Supplementary file 1 — (DOCX 889 KB) [file 11357_2025_1814_MOESM1_ESM.docx]

**Alterations in Mitochondria and Cellular Senescence in Aged sEH Null Female Kidneys**

Ala Yousef,^1^ Liye Fang,^2^ Mobina Heidari,^1^ Andy Huang,^1^ Patrick Kondraciuk,^2^ Kristen A. Yee,^2^ Michael Mengel,^3^ John M. Seubert^1,2*^

^1^Faculty of Pharmacy and Pharmaceutical Sciences, University of Alberta, Edmonton, Alberta, Canada

^2^Department of Pharmacology, Faculty of Medicine and Dentistry, University of Alberta, Edmonton, Alberta, Canada

^3^Department of Laboratory Medicine and Pathology, University of Alberta, Edmonton, Canada

**Supplementary data**

Table S1: qPCR Primer Sequences

| Target gene | Primer forward sequence (5’ to 3’) | Primer reverse sequence (5’ to 3’) |
| --- | --- | --- |
| *Nd1* | CTAGCAGAAACAAACCGGGC | CCGGCTGCGTATTCTACGTT |
| *Hk2* | GCCAGCCTCTCCTGATTTTAGTGT | GGGAACACAAAAGACCTCTTCTGG |
| *Mcp1* | ATGCAGGTCCCTGTCATG | GCTTGAGGTGGTTGTGGA |
| *Il-1β* | TGCCACCTTTTGACAGTGATG | GGAGCCTGTAGTGCAGTTGT |
| *Gapdh* | CTTTGTCAAGCTCATTTCCTGG | TCTTGCTCAGTGTTGC |
| *P16* | CGCAGGTTCTTGGTCACTGT | TGTTCACGAAAGCCAGAGCG |
| *P21* | CCTGGTGATGTCCGACCTG | CCATGAGCGCATCGCAATC |
| *P53* | CTCTCCCCCGCAAAAGAAAAA | CGGAACATCTCGAAGCGTTTA |
| *Kim-1* | ATGAATCAGATTCAAGTCTTC | TCTGGTTTGTGAGTCCATGTG |
| *18S rRNA* | TAGAGGGACAAGTGGCGTTC | CGCTGAGCCAGTCAGTGT |
| *zBP1* | TCAAAGGGTGAAGTCATGGA | GTGGAGTGGCTTCAGAGCTT |
| *Ifit-1* | CAAGGCAGGTTTCTGAGGAG | GACCTGGTCACCATCAGCAT |
| *Ifnb1* | CGTGGGAGATGTCCTCAACT | CTGAAGATCTCTGCTCGGACC |

**Figure S1:** Validation of fraction purity by immunoblotting. The same membrane was probed for GAPDH, a cytosolic marker, and VDAC-1, a mitochondrial outer membrane marker. VDAC-1 was detected only in the mitochondrial fraction, while its absence in the cytosolic fraction confirms minimal mitochondrial contamination, validating the reliability of cytosolic mtDNA measurements.


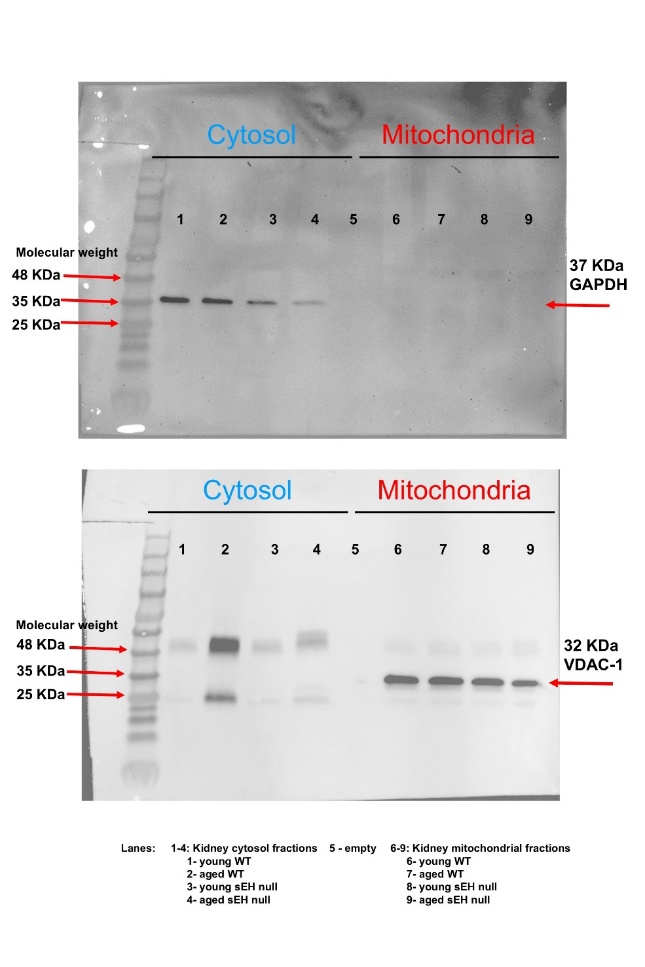


**Figure S2:** Representative images of periodic acid-Schiff (PAS) stained sections of young and aged WT and sEH null kidneys showing glomerular and tubular structures. Magnification: 20x and 63x objective lens and (10x) camera lens.

**
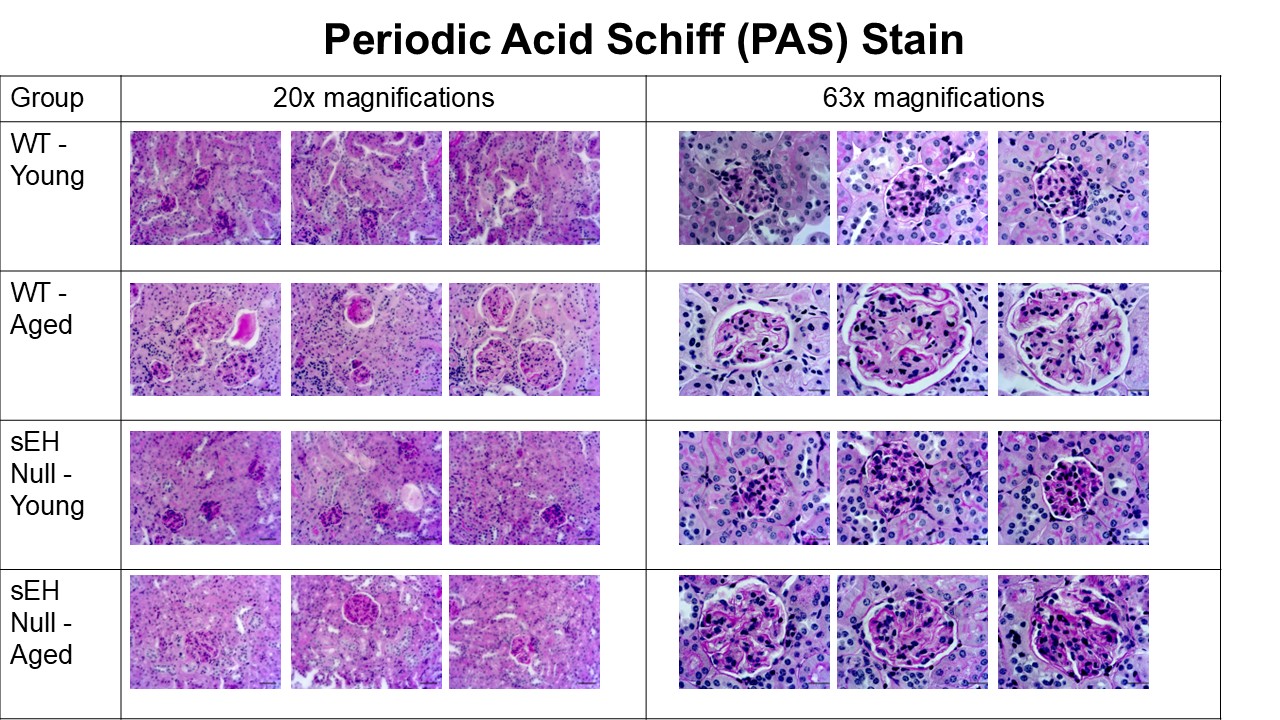
**

**
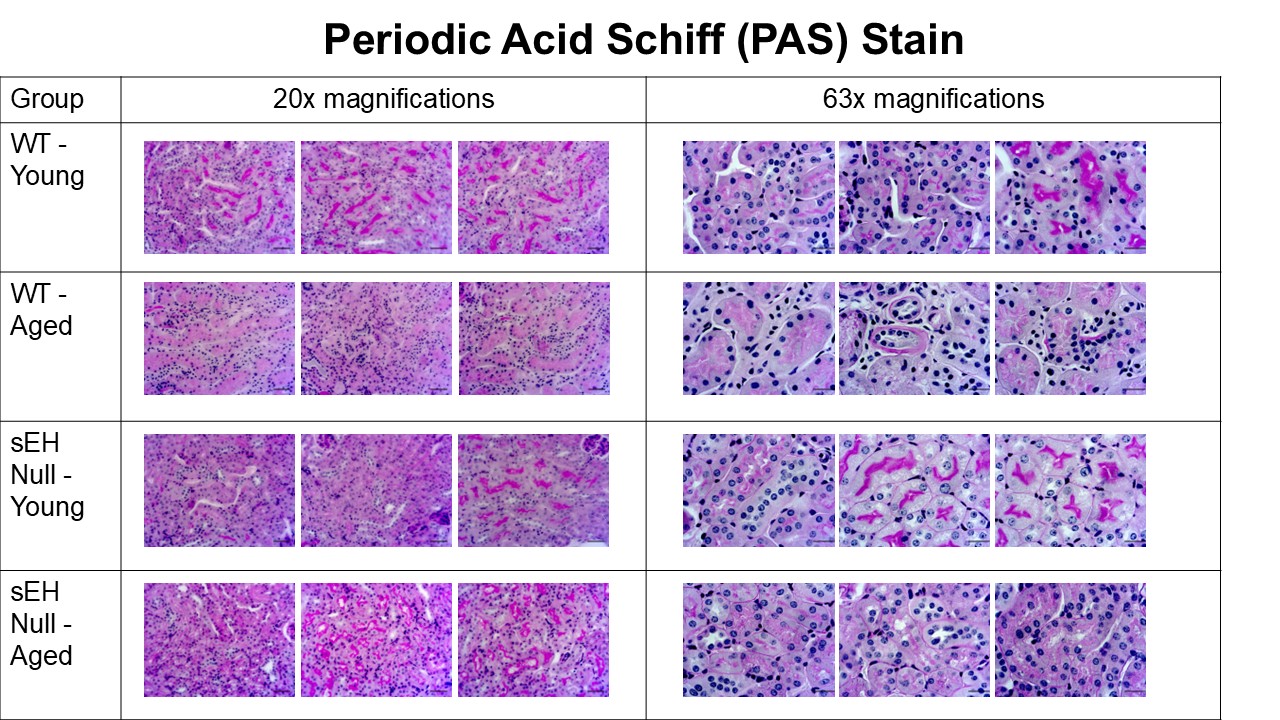
**

**Figure S3:** Mitochondrial function in young and aged kidneys from WT and sEH null mice. (A) Basal oxygen consumption rate (OCR) respiration. (B) ATP-dependent respiration. (C) Ratio of basal respiration OCR/citrate synthase activity. (D) Ratio of ATP-dependent respiration//citrate synthase activity. Data represented as mean ± SEM, *p*<0.05, (n=4-6). Statistical analysis was done using unpaired t-test.

**
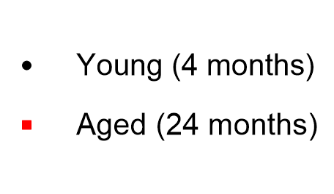
**

**
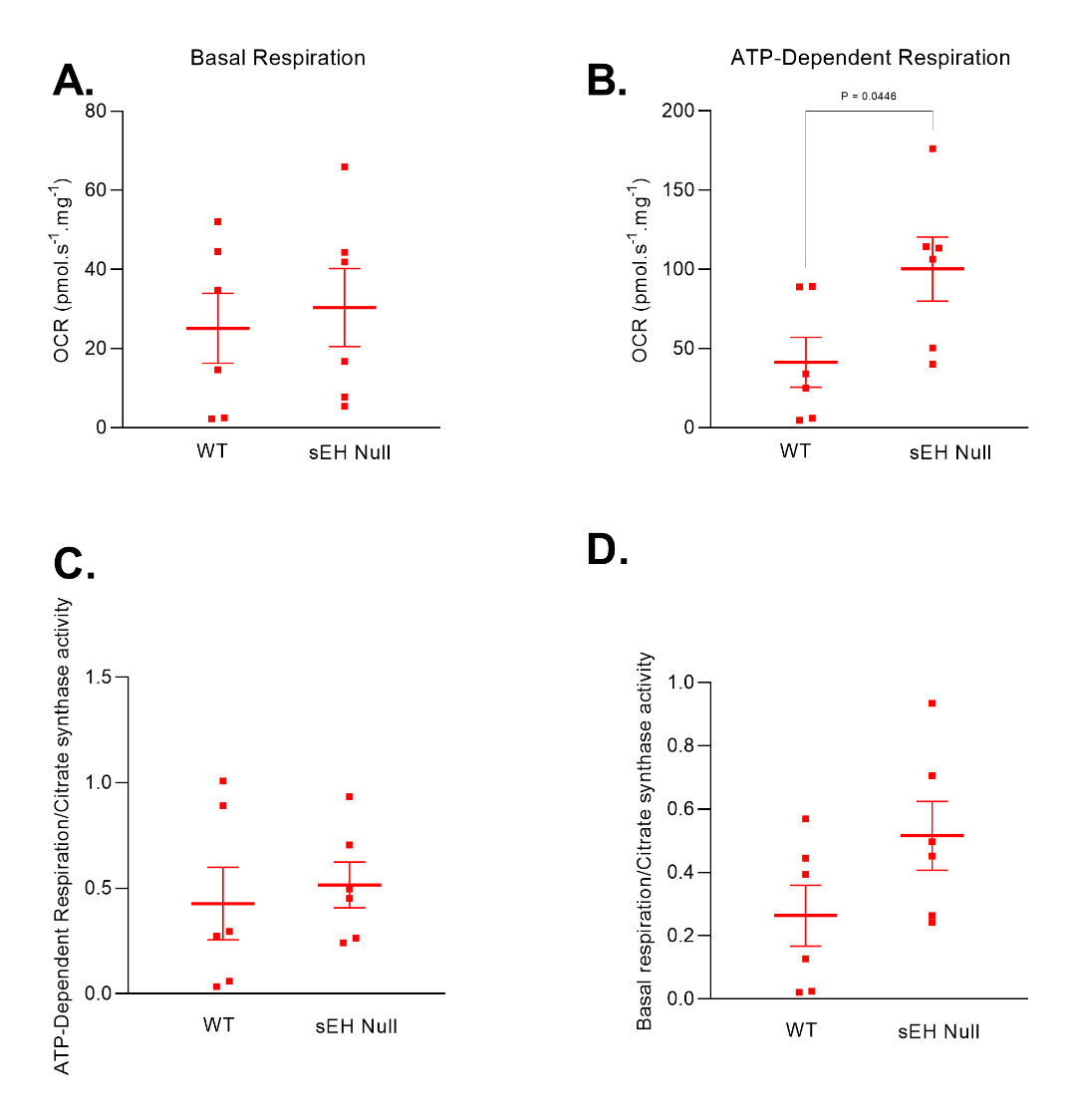
**
